# Correlating structure and photophysical properties in thiazolo[5,4-*d*]thiazole crystal derivatives for use in solid-state photonic and fluorescence-based optical devices†

**DOI:** 10.1039/d3ma00686g

**Published:** 2023-10-20

**Authors:** Abhishek Shibu, Sean Jones, P. Lane Tolley, David Diaz, Carly O. Kwiatkowski, Daniel S. Jones, Jessica M. Shivas, Jonathan J. Foley, Thomas A. Schmedake, Michael G. Walter

**Affiliations:** a Department of Chemistry, University of North Carolina at Charlotte Charlotte North Carolina 28223 USA Michael.Walter@charlotte.edu

## Abstract

There is a growing demand for new fluorescent small molecule dyes for solid state applications in the photonics and optoelectronics industry. Thiazolo[5,4-*d*]thiazole (TTz) is an organic heterocycle moiety which has previously shown remarkable properties as a conjugated polymer and in solution-based studies. For TTz-based small molecules to be incorporated in solid-state fluorescence-based optical devices, a thorough elucidation of their structure–photophysical properties needs to be established. Herein, we have studied four TTz-based materials functionalized with alkyl appendages of varying carbon chain lengths. We report the single crystal structures of the TTz derivatives, three of which were previously unknown. The packing modes of the crystals reveal that molecular arrangements are largely governed by a chorus of synergistic intermolecular non-covalent interactions. Three crystals packed in herringbone mode and one crystal packed in slipped stacks proving that alkyl appendages modulate structural organization in TTz-based materials. Steady state and time-resolved photophysical properties of these crystals were studied *via* diffuse-reflectance, micro-Raman, and photoluminescence spectroscopy. The crystals fluoresce from orange-red to blue spanning through the whole gamut of the visible spectrum. We have established that photophysical properties are a function of crystal packing in symmetrically substituted TTz-based materials. This correlation was then utilized to fabricate crystalline blends. We demonstrate, for the first time, that symmetrically substituted donor–acceptor–donor TTz-based materials can be used for phosphor-converted color-tuning and white-light emission. Given the cost effectiveness, ease of synthesis and now a structure–photophysics correlation, we present a compelling case for the adoption of TTz-based materials in solid-state photonic and fluorescence-based optical devices.

## Abstract 2

In thiazolo[5,4-*d*]thiazole (TTz)-based crystals, synergistic non-covalent interactions govern photophysical properties. Therefore, by modulating molecular-packing, TTz-based crystals can be tailored to fit optical and photonic applications such as white-light emissive organic phosphors.

## Graphical abstract

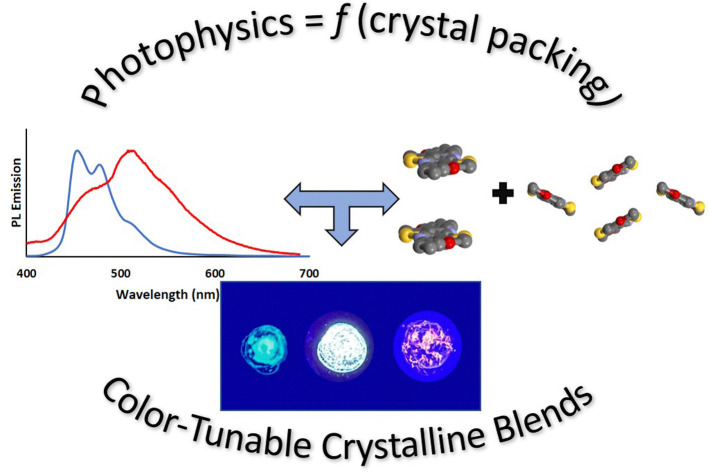

## 1. Introduction

In the last few decades, small organic molecular dyes have proven to be pivotal for a plethora of optical and photonic applications such as organic light emitting diodes, photovoltaics, sensors, dye mediated phototherapeutics, *etc.*^1–10^ The economies built around these technologies depend heavily on the discovery and development of new dyes for its sustained growth. For example, there is presently a strong demand for organic phosphor-converted (pc) white-light-emitting-diodes (WLED) in the solid-state lighting industry.^11–14^ Ideal candidates for this application would exhibit high fluorescence, high thermal and moisture stability, inexpensive processability and should be free of rare-earth-elements. Thiazolo[5,4-*d*]thiazole (TTz) is a heterocycle moiety which exhibits all these characteristics.^15–18^ TTz-based materials are inexpensive and facile to synthesize. Their strong electronegativity renders a high level of oxidative stability. They are thermally stable above 200 °C and it is easy to functionalize these materials with a broad array of groups to modulate their photophysical properties. Due to their versatile tunability, they have been previously studied in the context of voltage sensitive dyes, solar cells, sensors, and metal–organic framework mediated photon up-conversion.^19–22^ However, to our knowledge, no attempts have been made to utilize TTz based materials for organic pc-WLED applications. This is arguably because there remains much to be understood regarding excited-state energy (exciton) management in these dyes particularly as symmetrically substituted donor–acceptor–donor (D–A–D) based materials in the solid state.

A variety of TTz-containing polymeric materials have been studied for photocatalysis, electrical conductivity, and solar cell applications showing versatile photophysical properties, strong electron withdrawing capabilities and high charge mobilities.^23–28^ Exciton management in small molecule symmetrically substituted D–A–D-based TTzs has however been relatively underexplored. If small molecule symmetrically substituted D–A–D-based TTz dyes are to be integrated in photonic and optical devices on a mass scale, a thorough elucidation of their structure and photophysical properties in the solid state is required. This is a challenging task since aggregation-caused-quenching-effects and many-body-effect complicate the study of exciton dynamics and structure–photophysical property relationships in solid-state materials.^29^ Although few groups have reported on this relationship in the past utilizing D–A–D TTzs, a methodical study with a high degree of control over this correlation is yet to be established.^30,31^ To address this gap of knowledge of exciton management in solid-state symmetrically substituted D–A–D TTzs, we strategically synthesized four symmetrically substituted TTz-based materials functionalized with alkyl appendages of varying lengths. Since photophysical properties, such as singlet radiative recombination rates in the solid state, is a function of neighboring transition dipole orientation and distance, it was crucial to resolve the packing in crystals.^32^ We have addressed three questions: (1) will variations in alkyl chain lengths modulate molecular packing in TTz-based crystals? (2) will modulation of molecular packing influence photophysical properties like exciton recombination lifetime and photoluminescence emission? (3) can these crystals be utilized for color-tuning and pc-white-light emission applications in the solid state? herein, we report their crystal structures, molecular packing, steady state, and time-resolved photophysical properties. We have also studied crystalline blends of these materials for color-tunability in phosphor-converted light emission applications. Lastly, we report white-light emissive crystalline mixtures utilizing alkoxyphenyl TTz blends.

## 2. Results and discussion

**Fig. 1:**
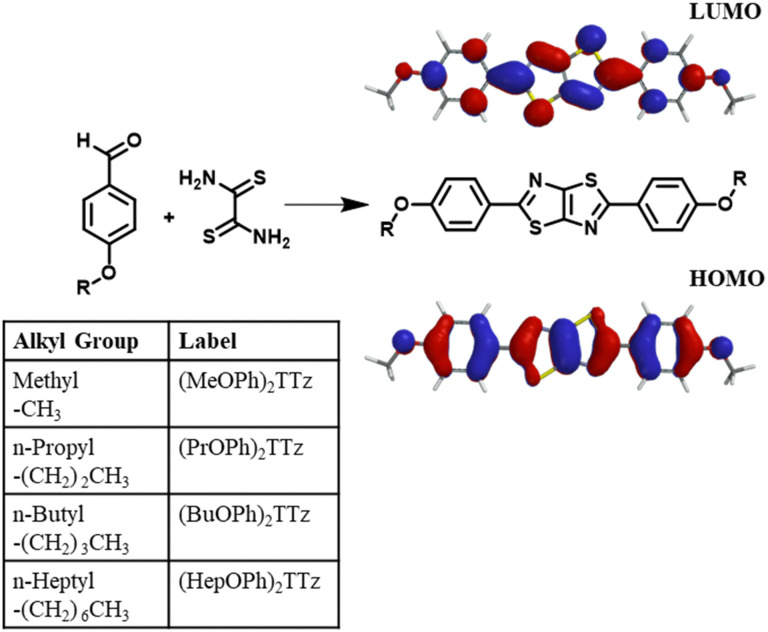
Schematic for the synthesis of dialkoxyphenyl thiazolo[5,4-*d*]thiazole is shown above along with electrostatic potential maps of ground state (HOMO) and first singlet excited state (LUMO) emphasizing the D–A–D character.

To study exciton management in D–A–D solid-state TTz-based materials, we functionalized the TTz core with symmetric *para*-substituted dialkoxyphenyl groups with varying alkyl chain lengths (Fig. 1).

The π conjugation of the electron acceptor TTz central heterocycle moiety is further extended by the phenyl groups on either side. This extended conjugated backbone broadens the ensemble of excited states into a quasi-continuous ‘band’ leading to increased probability density of excitons diffused over individual chromophores. This extended excited state band will influence excitonic coupling with neighboring molecules in condensed matter states *via* non-covalent intermolecular interactions.^29^ Alkoxy appendages would serve as electron donor groups (Fig. 1). Alkyl appendages of varying lengths are remarkable candidates for use as donor groups to explore exciton manipulation.^33–37^ Variation in their lengths has negligible effect on the electronics of the molecule. However, they have been previously shown to influence intermolecular packing in the solid state.^38–41^ This unique property of alkyl groups makes them highly suitable electron donors when establishing excitonic characteristics of a central organic moiety in solid-state materials.

### 2.1. Structural studies

#### 2.1.1. Single crystal XRD studies

Single crystals of (MeOPh)_2_TTz were obtained and isolated after rinsing the reaction product repeatedly with ethanol and water. The crystal structure was compared with previously reported literature and found to be consistent.^42^ We report crystallographic data for single crystals of (PrOPh)_2_TTz, (BuOPh)_2_TTz, and (HepOPh)_2_TTz for the first time. Single crystals of (PrOPh)_2_TTz, and (HepOPh)_2_TTz were grown through solvent–vapor diffusion technique with hexanes and chloroform. Single crystals of (BuOPh)_2_TTz were grown *via* solvent–liquid–liquid–diffusion method with hexanes and chloroform. The crystals were characterized using single crystal X-ray diffraction to obtain the molecular packing in these materials. The crystal parameters are summarized in Table S1 (ESI†). Significant alkyl chain dependent variations were observed in the packing of these crystals. These variations could strongly influence excitonic behavior in these materials.

The crystals packed in either monoclinic (*P*2_1_/*c*) or triclinic (*P*1̄) unit cell. Interestingly, the crystals that packed in monoclinic type showed a perfect correlation between volume of unit cell and alkyl chain length, (Fig. S2, ESI†). Crystallographic data reveals high degree of planarity in all molecules except in the case of (PrOPh)_2_TTz where the final carbon atom on the alkyl appendage is turned 113° out of the TTz plane following a trans conformation.

#### 2.1.2. Crystal packing motifs

**Fig. 2:**
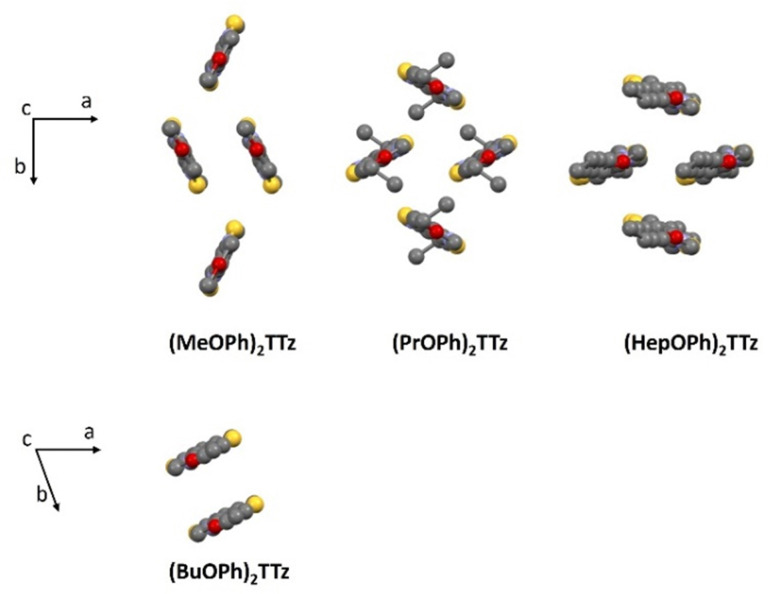
Crystal packing diagram of (C_*n*_OPh)_2_TTz unit cell. Top panel: Herringbone packing mode series; Bottom panel: slipped stack packing mode. H atoms are omitted for clarity.

**Fig. 3:**
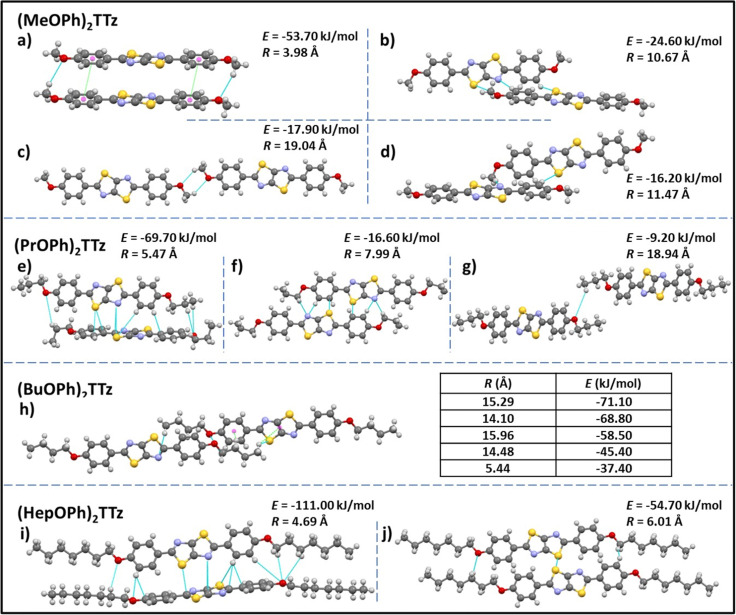
Unique intermolecular non-covalent interactions and orientation among (C_*n*_OPh)_2_TTz extrapolated from crystal packing and modeling studies. *E* is the total energy of interaction among the molecular pairs. The distance between molecular centroids is denoted by *R*. (*cf.* Tables S2, S3 and Fig. S3, ESI†).

Intermolecular interactions and arrangements typically drive energy fluxes and consequently, photophysical properties of bulk organic crystals. Therefore, insights into crystal packing motifs are useful predictors of excitonic behavior in organic solid-state materials.^43,44^Fig. 2 contains the unit cell molecular packing diagram of the four TTz crystal derivatives tilted along *c* axis.

(MeOPh)_2_TTz, (PrOPh)_2_TTz, and (HepOPh)_2_TTz arranged in herringbone pattern while (BuOPh)_2_TTz arranged in slipped stacks. SC-XRD packing data was used in conjunction with CrystalExplorer17 software package at B3LYP/6-31G(d,p) level to aid in establishing which intermolecular interactions would influence energy flux in the TTz crystals.^45^ Fig. S3 (ESI†) depicts color-coded geometry-based pictorial representation of interaction energies in TTz crystals with respect to one central reference molecule. Details of these interaction energies have been enumerated in Table S2 (ESI†). Based on the experimental and modeling data, molecular pairs of distinct geometric arrangements were extrapolated and have been shown in Fig. 3 with emphasis on the intermolecular interactions.

All the intermolecular interactions from crystal packing studies have been enumerated and discussed further in Table S3 (ESI†). In the case of (MeOPh)_2_TTz four unique molecular orientations can be observed. The cofacial arrangements (Fig. 3a and c) have close C–H–O interactions at 2.9 and 2.5 Å, respectively. In Fig. 3a, the molecules align similar to an H-type aggregate with a slipping angle of 101.93°. The TTz cores and phenyl rings stack on top of each other in this arrangement. The centroids of the phenyl rings are 3.9 Å apart. The π–π stacking arrangement could be the reason why this pair has the highest total energy of interactions in this crystal (−53.70 kJ mol^−1^) with the highest contribution from dispersion energy component. There are two orthogonal arrangements in the (MeOPh)_2_TTz crystal, (Fig. 3b and d). In both these arrangements, the alkyl chain and phenyl ring interact with the π conjugated TTz core. In (PrOPh)_2_TTz crystal, molecules arrange in three conformations. Among these, the orthogonal orientation (Fig. 3e) has the highest number of possible interactions with contacts distributed throughout the molecule. This arrangement also has the highest number of pairs (*N* = 4), and the highest energy of interactions (−69.70 kJ mol^−1^) in this crystal. Most of these contacts are interacting with the TTz core. One of the two cofacial arrangements (Fig. 3f) has multiple interactions with the TTz core as well. Also, among the orthogonal (Fig. 3e) and cofacial (Fig. 3g) arrangements there are multiple C–H–O interactions present. In the case of (BuOPh)_2_TTz crystal, the alkyl appendages are responsible for all the interactions. The molecules arrange in J-type cofacial slipped stacks, Fig. 3h, with C–H–(O, N, S, π_phenyl centroid_, and TTz_centroid_) interactions. The proximity of the molecules and the uniform cofacial arrangement results in very similar total energy of interactions among the pairs (−37.40 to −71.10 kJ mol^−1^) with the highest contribution from the dispersion energy. In (HepOPh)_2_TTz crystal, the orthogonal arrangement (Fig. 3i) is the most stable pair among all the molecular pairs depicted in Fig. 3 with total energy of interactions at −111 kJ mol^−1^. This could be because there are multiple interaction sites throughout the molecule pair. (HepOPh)_2_TTz also has a cofacial pair with C–H–O and S–S interaction.

**Fig. 4:**
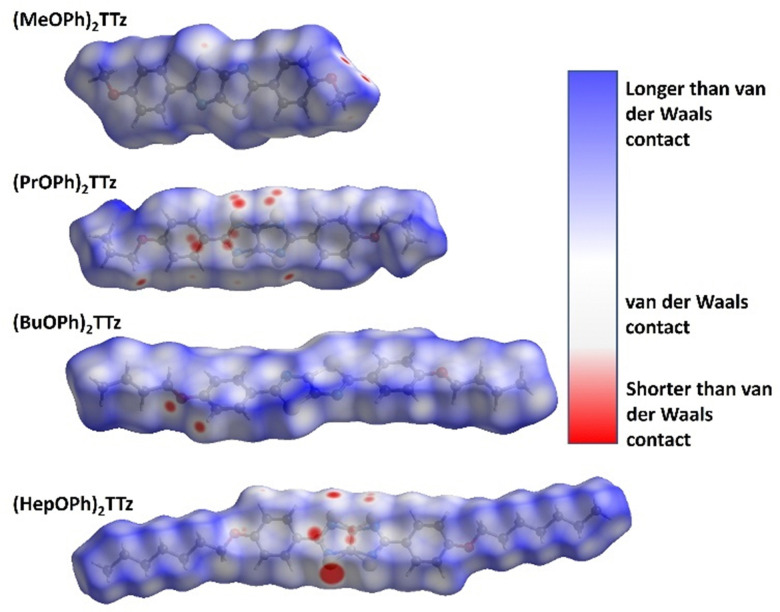
Normalized *d*_norm_ mapping images of the Hirshfeld surface analysis of the TTz crystals.

It is not enough to establish all the non-covalent intermolecular contacts in a crystal. Intermolecular interactions are inherently competitive. Since the aim of this study is to establish structure–property relationship in TTz crystals, it is important to ascertain which interactions would dominate the crystal packing. To address this problem, we simulated Hirshfeld surfaces of the molecules in the crystal unit cell. Hirshfeld surface simulations quantifies the intermolecular atomic contacts in the crystal using the descriptor *d*_norm_. The normalized interatomic contact distance (*d*_norm_) accounts for contact distances between nearest atoms present inside (*d*_i_) and outside (*d*_e_) the simulated surface and is expressed using eqn 1.1
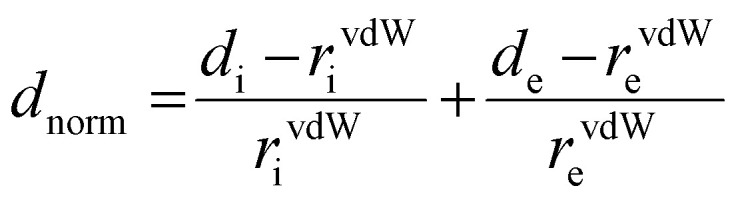
Here, *r*^vdW^_i_ and *r*^vdW^_e_ represent the van der Waals radii of the atoms internal and external to the surface, respectively. The Hirshfeld surface simulations rendered *d*_norm_ images with fixed color scale of −0.0955 (red) to 1.1742 (blue), shown in Fig. 4. The images utilized a red-white-blue color scheme where red highlights the shorter contacts, white is used for interactions around the vdW separation distance, and blue is for longer contact distances. The red spots become brighter and bigger as the internuclear distances decrease.

All the types and distribution of relative contributions of intermolecular contacts to the Hirshfeld surface area for the (C_*n*_OPh)_2_TTz crystals have been provided in Fig. S5 (ESI†). In Fig. 4a, significant interaction sites near the alkyl appendage and the oxygen atom of the (MeOPh)_2_TTz molecule is observed. These sites represent the C–H–O bonding which was depicted in the cofacially arranged molecules in Fig. 3a and c. There is also a faint interaction site visible at the TTz core. This interaction is caused by the orthogonal arrangements in the crystal. In the case of (PrOPh)_2_TTz, many interaction sites are visible. Most of these sites are localized around the TTz core and phenyl rings indicating that the π conjugated backbone of the molecule is well connected with other molecules within the crystal. The most dominant interaction in (BuOPh)_2_TTz is the C–H–O contact. This interaction is established between the phenyl ring and oxygen atom of neighboring molecules. The TTz core of (HepOPh)_2_TTz molecule is strongly interacting with the orthogonal and cofacial molecules. The big red spot on the TTz core is indicative of the many sulfur-based contacts in this crystal.

A salient packing-dependent feature of organic solid-state materials that influences photophysical and excitonic properties is π–π stacking. The Shape Index feature of Hirshfeld surfaces was utilized to ascertain the presence of π–π stacking in the (C_*n*_OPh)_2_TTz crystals. The results are shown in Fig. S6 (ESI†). (MeOPh)_2_TTz crystals show evidence for π–π stacking around the phenyl rings. The stacking is caused because the phenyl centroids of cofacial (MeOPh)_2_TTz molecules interact with each other in a crystal environment. It must be noted that (MeOPh)_2_TTz was the only crystal among the four TTz derivatives in this study that exhibited pronounced π–π stacking. (BuOPh)_2_TTz exhibited signs of stacking along the alkyl chain. These were caused by C–H–π interactions.

Based on the single-crystal X-ray diffraction experiments and computational modeling, we can arrive at the following conclusions about the crystal packing of (C_*n*_OPh)_2_TTz crystals and excitonic property predictions. The cofacial molecular pair in (MeOPh)_2_TTz crystal is the most stable intermolecular configuration in this crystal with high dispersion energy. This pair also has significant π–π stacking which could lead to exciton formation diffused over multiple molecules. Such geometry-energy related effects could result in significantly red-shifted PL emission spectra and long singlet lifetimes.^34^ The π conjugated backbone of (PrOPh)_2_TTz is well connected to other molecules in the crystal. The most stable of these interactions are with orthogonally oriented molecules. This could lead to a broad PL emission spectrum and release of low energy photons. The (BuOPh)_2_TTz arranged in J-type cofacial slipped stacks with most interactions limited to the alkoxy appendages. The similar total energy of interactions among the molecular pairs along with the high contribution from dispersion energy component indicates to the possibility of exciton delocalization over multiple molecules and relaxation of excited energy *via* non-radiative pathways. Therefore, it can be predicted that the PL emission spectral shape and singlet radiative recombination rates of (BuOPh)_2_TTz crystal would resemble its solution counterpart while non-radiative pathways could lead to loss of luminescence efficiency. The TTz core of (HepOPh)_2_TTz is connected through multiple contacts with neighboring orthogonally oriented molecules. These interactions have the highest total energy among the four TTz crystal derivatives and thus would play the dominant role in excitonic properties of this crystal. The orthogonal orientation would result in a broad ensemble of excited state bands which could encourage exciton delocalization. Such delocalization could result in long exciton lifetimes, broad PL emission profile, and release of low energy photons. Crystallographic studies thus conclusively prove that small variations in alkyl chain length, a rather understated donor group, leads to a wide range of modulation in molecular packing in TTz-based crystals.

### 2.2. Photophysical studies

#### 2.2.1. Steady state photophysical studies

**Fig. 5:**
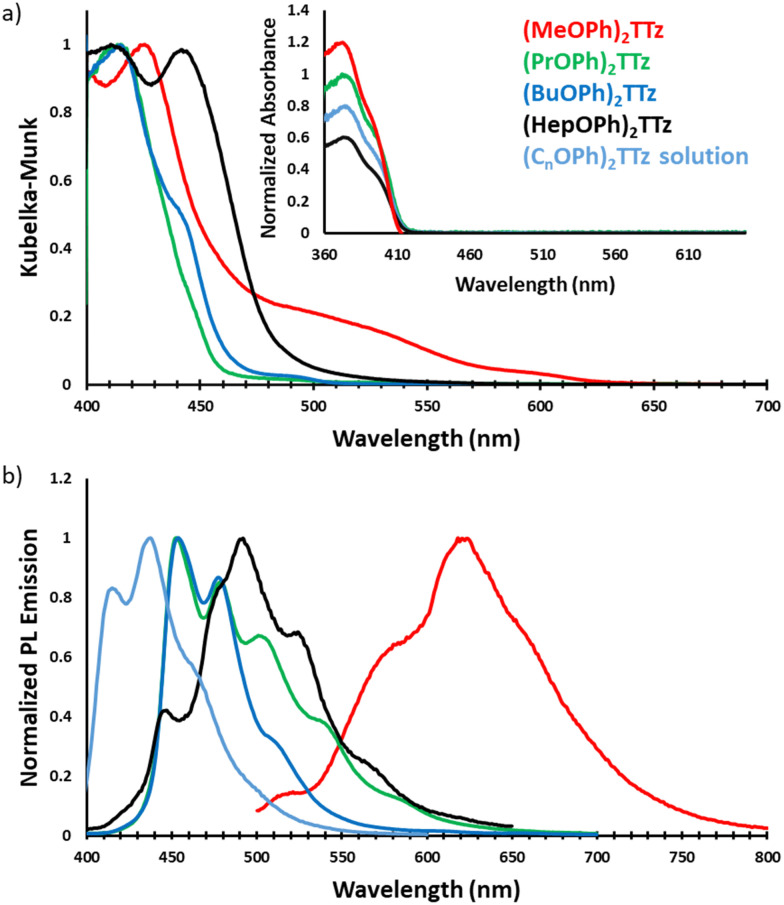
Steady state photophysical characteristics of symmetrically substituted dialkoxyphenyl TTz crystals. (a) Absorption spectra of TTz crystals obtained *via* diffuse reflectance spectroscopy. Inset: UV-Vis absorption spectra of TTzs in solution state. (b) Photoluminescence spectra of TTz crystals and solution.

In solution state, the alkyl appendages have no effect on the photophysical properties of the TTz derivatives (inset Fig. 5). Also, all the derivatives exhibit approximately the same quantum yield in solution state as well (*Φ*_F_ = 25–28%). This is expected since the length of the alkyl chains have negligible contribution to the intrinsic electronic properties of the molecule. However, in the solid state, the alkyl chains strongly modulate the photophysical properties of these derivatives. Fig. 5 shows the absorption and PL emission characteristics of the crystal derivatives.

TTz-based symmetrically substituted crystals have significantly red-shifted spectra when compared to their solution counterparts. They also exhibit wide variance in their absorption characteristics when compared to each other. These features can be attributed to the unique intermolecular transition dipole orientation and arrangement previously elucidated from crystal packing studies. Since many molecules in a crystal interact with an external field through their local polarizabilities, the energy of each molecular pair established above was considered, Table S2 (ESI†). As shown earlier, (MeOPh)_2_TTz has 4 unique molecular pair arrangements. The orthogonal pair depicted in Fig. 3d has the lowest energy, −16.20 kJ mol^−1^. This would explain why (MeOPh)_2_TTz crystal has the smallest bandgap. The highest polarization energy in this derivative was represented by the cofacial pair depicted in Fig. 3a, −53.70 kJ mol^−1^. This wide range of energies exhibited by the molecular pairs of this crystal could explain the broad absorption spectral profile. The three remaining crystal derivatives have at least 1 pair with high energy of >70 kJ mol^−1^. In the presence of such strongly polarizable pairs, signals from other pairs could be suppressed. Interestingly, a shoulder in the case of (BuOPh)_2_TTz at 443 nm can be observed which could be indicative of strong excitonic coupling within the intermolecular planes. For (HepOPh)_2_TTz, the peak at 416 nm is caused by the cofacial molecular arrangement depicted in Fig. 3j. However, it must be noted that the orthogonal arrangement shown in Fig. 3i is the most energetically stable pair not just in this crystal, but among all the arrangements established in this study, and hence could be responsible for the strong transition at 444 nm leading to the broad spectra and second smallest excitonic bandgap in the TTz crystal series.

**Fig. 6:**
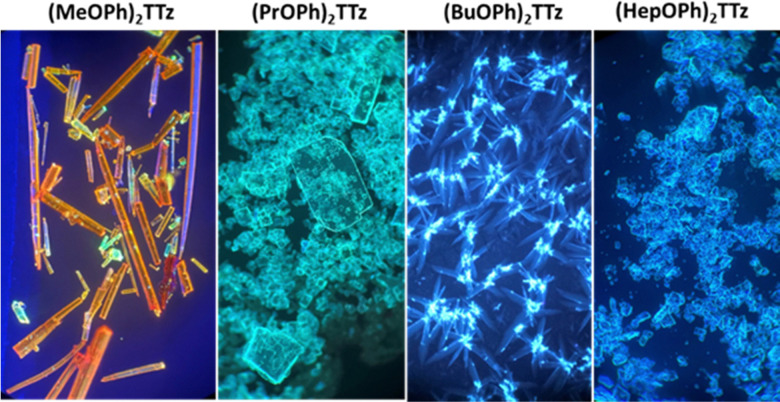
Images of (C_*n*_OPh)_2_TTz illuminated by UV lamp (400 nm) and viewed under an optical microscope at 10× magnification.

Images of the crystals are shown below (Fig. 6).

All TTz crystals have red-shifted PL emission when compared to the solution state. (PrOPh)_2_TTz and (BuOPh)_2_TTz have the least red-shifted spectra. (BuOPh)_2_TTz has a relatively sharp emission profile, similar to TTz solution, and hence has blue emission in the solid state. (PrOPh)_2_TTz has a broad emission profile which renders the crystals cyan fluorescence. The secondary peaks and broad profile could be the result of the low photon energy release from the orthogonal molecular interaction. The effect of the orthogonal interaction is maximized in emission spectra of (HepOPh)_2_TTz. The transition at 450 nm typically seen among the cofacial pairs in this crystal series is suppressed by the peak at 495 nm. (MeOPh)_2_TTz has the most red-shifted spectra, and the crystals fluoresce orange-red. This red emission could be due to the weak polarization energy discussed earlier. Among all molecular arrangements established in this study, (MeOPh)_2_TTz has the most easily polarizable pairs. The orthogonal molecular arrangement depicted in Fig. 3d could be responsible for the drastic red-shift in this crystal. Recently, Wei *et al.* reported on the origin of red shifted transition in a very similar TTz-based crystal.^46^ They propose that the shift is caused mainly due to excitation polarization of unparalleled aggregates. This interpretation would be in congruence with our hypothesis as well. The wide overlap between the absorbance and emission spectrum of (MeOPh)_2_TTz could be caused due to anisotropy and is typical with organic crystals.^47^ The green emission in the crystal image was maximized by the peak at 525 nm in the emission profile. This is due to surface-to-kernel effect where surface energy perturbs the crystal packing, inducing defects-related-effects.^46,48^

**Fig. 7:**
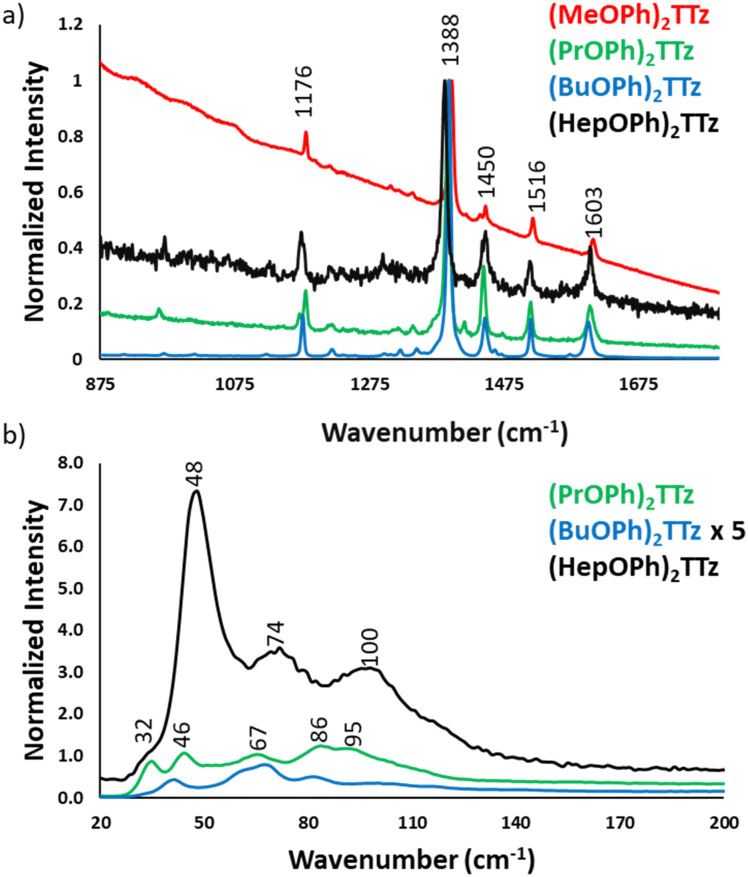
Micro-Raman spectra of TTz crystals.

To further investigate if modulation of molecular packing in crystals would influence the excited state photophysical properties, the TTz crystals were characterized using micro-Raman spectroscopy, Fig. 7.

Since the central diphenyl TTz moiety is common in all the derivatives, the spectral signature in the fingerprint region, 875 to 1800 cm^−1^, is the same in all the crystals, Fig. 7a. The experimental data was verified with DFT simulation and peak assignments have been shown in Fig. S8 (ESI†). It is, however, interesting to note the difference in the intensity of the spectra. (BuOPh)_2_TTz exhibited the highest signal to noise ratio with sharp and highly resolved peaks. This was expected since the packing studies revealed that intermolecular interactions are limited to the alkoxy region in this crystal. The crystals that packed in herringbone pattern displayed peaks at much lower intensity.

The region of particular interest lies from 10 to 200 cm^−1^, Fig. 7b. This region represents lattice-phonon vibration frequencies and has been probed in various studies to identify intermolecular packing dependent effects on photophysical properties in organic crystals.^49–52^ The low-energy modes are highly sensitive to molecular packing, which explains the observed difference shown above. All the spectra have been normalized by maintaining the C=N vibration of the thiazole ring at 1388 cm^−1^ constant. Interestingly, (BuOPh)_2_TTz, which displayed the most intense spectral features in the >800 cm^−1^ region had highly muted signals in the <200 cm^−1^ region. The spectrum was amplified by a factor of 5 in the interest of clarity. This further contributes to the packing dependent studies revealing that the intermolecular interactions are localized in the alkoxy appendage. The weak lattice-phonon vibrations juxtaposed against the sharp, unperturbed spectral features from the conjugated backbone would support the prediction that (BuOPh)_2_TTz crystal would exhibit excitonic characteristics similar to its solution counterpart. (HepOPh)_2_TTz crystal did not exhibit highly sharp features in the >800 cm^−1^ region. However, it has the strongest lattice vibration in the sub 200 cm^−1^ region with three clear bands. This remarkably intense spectral feature in the weak-energy modes indicates that excited states in this material would prefer to relax *via* lattice-phonon vibrations leading to low exciton radiative rates and poor fluorescence efficiency. (PrOPh)_2_TTz exhibited the second most intense spectral feature in the sub 200 cm^−1^ region. Most of the bands are similar to (HepOPh)_2_TTz, albeit in different relative ratio. This was expected since both monoclinic crystals pack in herringbone pattern but display subtle differences in intermolecular interaction types and energies. Unfortunately, (MeOPh)_2_TTz did not yield any resolvable spectral feature in the lattice vibration region, Fig. S9 (ESI†). Nevertheless, it is clear that the alkyl appendages modulate the steady state photophysical properties of these crystals drastically.

#### 2.2.2. Time-resolved photophysical studies

**Fig. 8:**
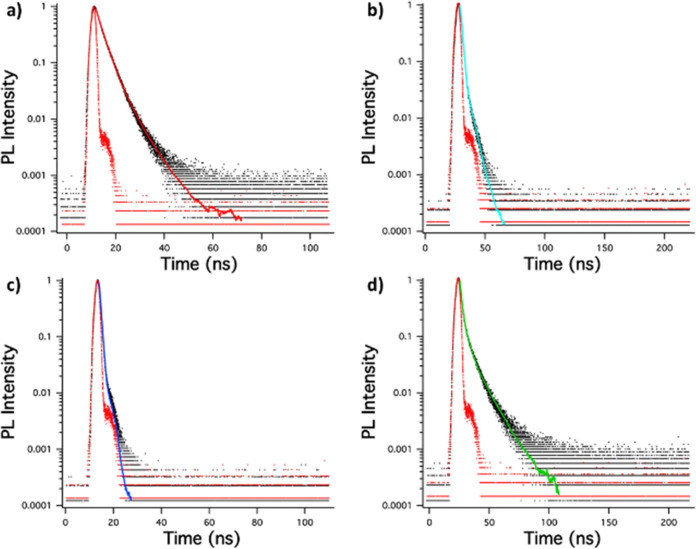
Singlet emission lifetime decay of (a) (MeOPh)_2_TTz, (b) (PrOPh)_2_TTz, (c) (BuOPh)_2_TTz, (d) (HepOPh)_2_TTz crystals.

The singlet emission lifetime decays of these crystals were measured and shown in Fig. 8.

It is interesting to note that the crystals exhibited much longer lifetimes than their solution counterparts. This is expected since bulk organic aggregates require higher reorganization energy than molecular monomers in dilute solutions.^29^ The fluorescence decay lifetime of the TTz based crystals was used along with their quantum yield to ascertain luminescence efficiency. This has been shown in Table 1 along with excitonic bandgap of the materials derived from absorbance Tauc plot.

**Table 1:** Excitonic bandgap values, luminescence efficiency data of the symmetrically substituted TTz crystals and solution

Material	*E*_g_ (eV)	*Φ*_F_ (%)	*τ*_avg_ (ns)	*k*_r_ (s^−1^)	*k*_nr_ (s^−1^)
(MeOPh)_2_TTz	2.46	10	3.35	2.99 × 10^7^	2.69 × 10^8^
(PrOPh)_2_TTz	2.73	27	1.66	1.63 × 10^8^	4.40 × 10^8^
(BuOPh)_2_TTz	2.74	4	0.67	6.43 × 10^7^	1.43 × 10^9^
(HepOPh)_2_TTz	2.52	0.4	2.34	1.58 × 10^6^	4.26 × 10^8^
(C_*n*_OPh)_2_TTz (solution)	2.98	28	0.54	5.19 × 10^8^	1.33 × 10^9^

(MeOPh)_2_TTz crystal exhibited the longest singlet lifetime in the series. This could be because of the pi–pi stacking revealed from the Hirshfeld analysis. Another reason for the long lifetime could be the low polarization energy of the molecular pairs in this crystal. (HepOPh)_2_TTz crystal has the second longest singlet lifetime, however, it also has the lowest *Φ*_F_. This could be because the highly energetically stable molecular pairs facilitate exciton delocalization diffused over multiple molecules in the crystal. (PrOPh)_2_TTz has the shortest singlet lifetime in the herringbone packing series. However, it has a remarkably high *Φ*_F_. The radiative rate in this crystal is comparable with its solution counterpart which is rare among similar molecular systems. The high radiative rates could be because all the intermolecular interactions in this derivative are centered around the TTz heterocycle. The slipped stacked (BuOPh)_2_TTz exhibited radiative decay lifetime similar to its solution state. This could be because of the limited geometry-energy related effects in this derivative. However, the *Φ*_F_ and *k*_nr_ suggest that the stacking localized around the alkoxy appendages in this J-type aggregate facilitates exciton delocalization.

Clearly, alkyl appendages induced structural modifications in symmetrically substituted D–A–D TTz-based crystals result in predictable modulation of photophysical properties. The change in photophysical properties is a function of molecular packing in these crystals. Having established a correlation between structure and photophysical properties in TTz-based crystals, we demonstrate the relevance of such a pedagogical study by fabricating novel TTz-based crystalline blends for all-organic phosphor-converted color-tuning and white-light emission applications.

### 2.3. CIE engineering

In the following section, the use of symmetrically substituted D–A–D dialkoxyphenyl TTz crystalline blends for generating a broad range of phosphor-converted light emission is demonstrated. We also prove that these crystalline blends can be used for generating white-light emission.

**Fig. 9:**
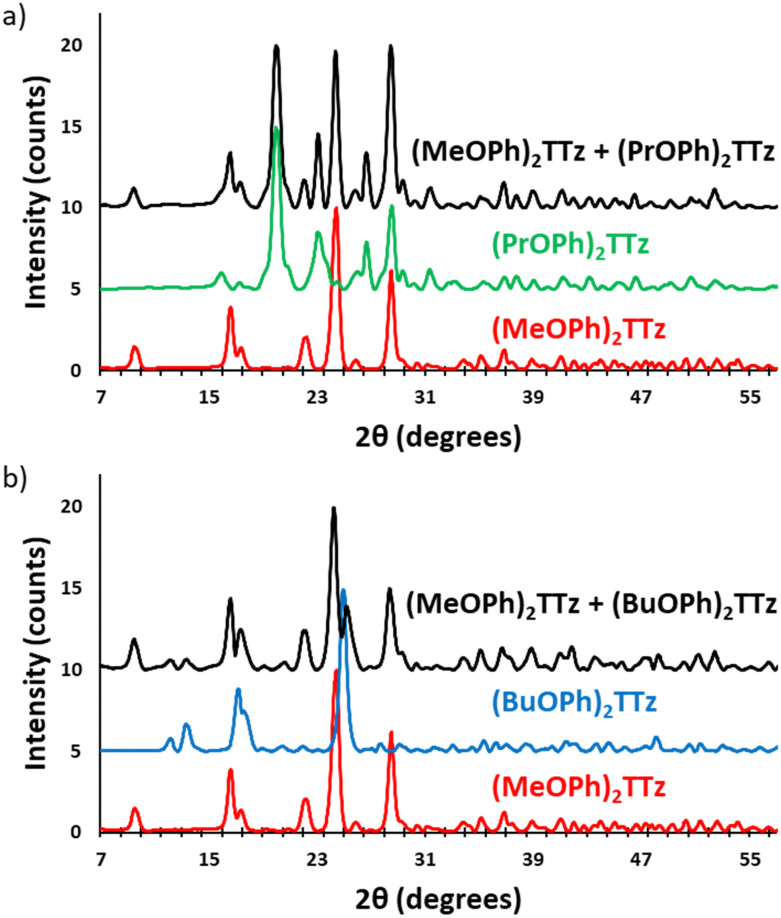
Powder X-ray diffractograms of parent TTz crystals and their blends. (a) Set 1: (MeOPh)_2_TTz, (PrOPh)_2_TTz, and (MeOPh)_2_TTz + (PrOPh)_2_TTz crystalline blend; (b) set 2: (MeOPh)_2_TTz, (BuOPh)_2_TTz, and (MeOPh)_2_TTz + (BuOPh)_2_TTz crystalline blend.

To determine if the crystalline blends are consistent with the single-crystal structural properties we have established above, the powder X-ray diffractograms of drop cast TTz crystals were compared with the powder diffractograms generated from the SC-XRD studies, (Fig. S7, ESI†). Since the XRD pattern of the drop cast crystals were found to be in strong agreement with the single-crystal studies, it stands to reason that they will also exhibit similar photophysical properties. Two sets of parent TTz crystals were chosen based on their diverse PL emission resulting in a potentially wide color-tuning window. The crystallinity of the resultant blends was investigated (Fig. 9).

The X-ray diffractograms are conclusive proof that the TTz blends have the crystalline properties of the parent TTz derivatives. Since photophysical properties are a function of crystal packing, excitons can be managed by modulating the parent TTz contribution in the blends. Therefore, the blend proportions were tuned and studied for color-tuning and white-light emission.

**Fig. 10:**
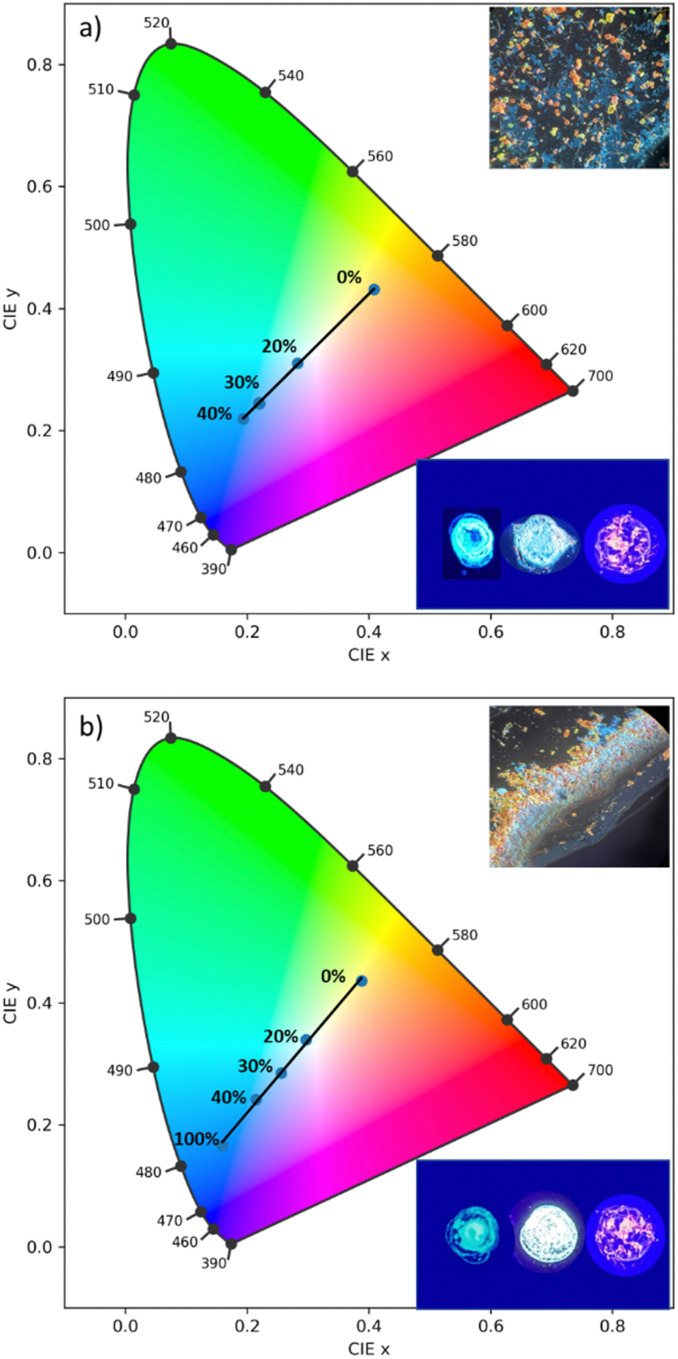
CIE plot establishing color-tuning capabilities of symmetrically substituted TTz-based dialkoxyphenyl crystals. (a) (MeOPh)_2_TTz crystals were mixed with (PrOPh)_2_TTz crystals at varying weight %. (b) Crushed (MeOPh)_2_TTz crystals were mixed with (BuOPh)_2_TTz crystals were mixed with (BuOPh)_2_TTz crystals at varying weight %. Selective weight proportions are plotted for clarity. Top inset: Optical microscopic image of crystalline admixtures at 4 : 1 wt% of the symmetrically substituted TTzs illuminated by 400 nm lamp and viewed at 10×. Bottom inset: Photographs of (PrOPh)_2_TTz/(BuOPh)_2_TTz (left); (MeOPh)_2_TTz (right); and multi-TTz crystalline white-light emissive blend illuminated by 400 nm lamp.

Utilizing the multi-fluorochromaticity of the symmetrically substituted dialkoxyphenyl TTz crystals, mixtures of the aggregates were drop cast on glass substrates from saturated DCM solutions with varying proportions by weight, (Fig. 10). In set 1 (Fig. 10(a)), orange-red emissive (MeOPh)_2_TTz crystals were mixed with varying proportions of cyan (PrOPh)_2_TTz crystal fluorophores. Set 2, (Fig. 10(b)), comprised of yellow fluorescent crushed (MeOPh)_2_TTz crystals with varying proportions of blue emissive (BuOPh)_2_TTz crystals.

The CIE plots establish that TTz crystals can be utilized for fabrication of multi-fluorochromic mixtures in the solid state. Increasing the contribution of (PrOPh)_2_TTz and (BuOPh)_2_TTz shifts the emission from (MeOPh)_2_TTz's orange-red to blue. This was consistent with our predictions based on the steady state photophysical properties established earlier in this study. The fluorescence of these TTz crystalline blends can be tuned to yield highly contrasting colors by just modulating the crystal concentration. The photostability of these crystals was tested under constant illumination for 5 hours, Fig. S10 (ESI†). The TTz crystals were found to be highly photostable even in ambient conditions. This additional feature strengthens the case of utilizing TTz-based materials for developing all-organic fluorescent phosphor layers with broad photoluminescence tunability. In both sets, mixtures with 4 : 1 ratio by weight yielded white-light emissive blends. The simplicity in fabricating these crystalline blends presents a compelling case for utilization of TTz-based materials for photonic and fluorescence-based optical device applications such as pc-color-tuning and white-light emission.

## 3. Experimental

### 3.1. Materials and instrumentation

4-*n*-Alkyloxybenzaldehyde and dithiooxamide were purchased from Sigma-Aldrich and used without further purification. Chloroform (CHCl_3_), dichloromethane (DCM), pentane, and hexanes were purchased from Alfa Aesar Chemicals and used without further purification. ^1^H-NMR measurements were carried out using JEOL 500 MHz NMR. Mass spectrometry measurements were obtained using a Perceptive Biosystems Voyager MALDI mass spectrometer. UV-Vis absorption spectrometric data for solution was obtained using Cary 300 UV-Vis spectrophotometer. Diffuse reflectance measurements of the crystals were collected using Cary 5000 UV-Vis spectrophotometer. Photoluminescence measurements were carried out on Jobin Yvon-Spex Fluorolog. PL decay lifetimes were obtained using a diode laser with a repetition rate of 1 MHz and excitation wavelengths of 389 nm. 9,10-Diphenylanthracene in cyclohexane was used as standard reference for quantum yield measurements.^53^ Solid state quantum yield measurements were carried out using QuantiPhi-2. Density functional theory (DFT) computational analysis were performed using Spartan '10 and CrystalExplorer17, at B3LYP/6-31G(D) and B3LYP/6-31G(d,p) level, respectively. The samples used for micro-Raman experiments were chosen with the aid of an optical microscope (Olympus BX41) interfaced to a Jobin Yvon XploRA PLUS Raman spectrometer, spanning the region 10–1800 cm^−1^. Excitation wavelength of 785 nm was chosen to avoid fluorescence. Neutral density optical filters were used to minimize the risk of crystal damage. Powder XRD *θ*/2*θ* patterns were obtained using ‘Xcalibur – Gemini ultra’ with Ni filtered Cu Kα radiation (*λ* = 1.541 Å) at 50 kV, 40 mA. The experiments were performed from 0° to 123.82° with 0.01 step size and a counting time of 1 s per point.

Single crystal X-ray diffractometry data were acquired with an Agilent (now Rigaku) Gemini A Ultra diffractometer. Single crystals of (MeOPh)_2_TTz were obtained and isolated after rinsing the reaction product repeatedly with ethanol and deionized water. The crystal structure was compared with previously reported literature and found to be consistent.^42^ Single crystals of (PrOPh)_2_TTz, and (HepOPh)_2_TTz were grown *via* solvent–vapor diffusion technique with hexanes and chloroform. Single crystals of (BuOPh)_2_TTz were grown using solvent–liquid–liquid–diffusion method with hexanes and chloroform. Single crystals of suitable size were coated with a thin layer of paratone-N oil, mounted on the diffractometer, and flash cooled to 100 K in the cold stream of the Cryojet XL liquid S55 nitrogen cooling device (Oxford Instruments). The diffractometer was equipped with sealed-tube long fine focus X-ray sources with Mo target (*λ* = 0.71073 Å) and Cu target (*λ* = 1.5418 Å), four-circle kappa goniometer, and CCD detector. Mo target was used to characterize (MeOPh)_2_TTz. Cu target was used for (PrOPh)_2_TTz, (BuOPh)_2_TTz, and (HepOPh)_2_TTz crystals. CrysAlisPro9 software was used to control the diffractometer and perform data reduction. The crystal structure was solved with SHELXS.^54^ All non-hydrogen atoms appeared in the E-map of the correct solution. Alternate cycles of model-building in Olex2 and refinement in SHELXL followed.^54,55^ All non-hydrogen atoms were refined anisotropically. All hydrogen atom positions were calculated based on idealized geometry and recalculated after each cycle of least squares. During refinement, hydrogen atom – parent atom vectors were held fixed (riding motion constraint).

### 3.2. Preparation of dialkoxyphenyl TTz derivatives

4-*n*-Alkyloxybenzaldyde was reacted with dithiooxamide in 10 mL of DMF for 24 h at 150 °C. The resulting precipitate was collected by filtration and rinsed with water and ethanol. The product was purified using silica gel column chromatography, and purity and structure were established using TLC, ^1^H-NMR, and MALDI-TOF mass spectrometry.

#### 3.2.1. 2,5-Bis(4-methoxyphenyl)thiazolo[5,4-*d*]thiazole ((MeOPh)_2_TTz)

The precipitate mixture was rinsed with ethanol and water repeatedly to yield 0.25 g (61.5%) of orange-red fluorescent needle-like crystals. ^1^H-NMR (500 MHz, CDCl_3_, TMS, *δ*): 7.93 (d, *J* = 1.98 Hz, 2H), 6.99 (d, *J* = 2.01 Hz, 2H), 3.88 (s, 3H). UV-Vis *λ*_max_ (CHCl_3_, *ε* = M^−1^ cm^−1^): 375 nm (*ε* = 27 816). MALDI-TOF-MS (calcd for C_18_H_14_N_2_O_2_S_2_): 354.44, found: *M* = 354.66. Quantum yield in CHCl_3_ (*λ*_ex_ = 375 nm, *λ*_em_ = 375 nm, *λ*_em_ = 435 nm) = 28%.

#### 3.2.2. 2,5-Bis(4-propoxyphenyl)thiazolo[5,4-*d*]thiazole ((PrOPh)_2_TTz)

Column chromatography using hexanes : DCM (1 : 1) afforded 0.45 g (47%) of product. ^1^H-NMR (500 MHz, CDCl_3_, TMS, *δ*): 7.92 (d, *J* = 1.98 Hz, 2H), 6.97 (d, *J* = 1.99 Hz, 2H), 3.99 (t, *J* = 2 Hz, 2H), 1.84 (m, 2H), 1.06 (t, *J* = 2.86 Hz, 3H). UV-Vis *λ*_max_ (CHCl_3_, *ε* = M^−1^ cm^−1^): 375 nm (*ε* = 70 183). MALDI-TOF-MS (calcd for C_22_H_22_N_2_O_2_S_2_, [M + H]^+^): 411.11, found: *M* = 411.06. Quantum yield in CHCl_3_ (*λ*_ex_ = 370 nm, *λ*_em_ = 437 nm) = 28%.

#### 3.2.3. 2,5-Bis(4-butoxyphenyl)thiazolo[5,4-*d*]thiazole ((BuOPh)_2_TTz)

Column chromatography using pentane : DCM (2 : 1) afforded 0.11 g (45%) of product. ^1^H-NMR (500 MHz, CDCl_3_, TMS, *δ*): 7.92 (d, *J* = 2 Hz, 2H), 6.98 (d, *J* = 2.04 Hz, 2H), 4.03 (t, *J* = 2 Hz, 2H), 1.80 (m, 2H), 1.52 (m, 2H), 0.99 (t, *J* = 3 Hz, 3H). UV-Vis *λ*_max_ (CHCl_3_, *ε* = M^−1^ cm^−1^): 375 nm (*ε* = 2 000 000). MALDI-TOF-MS (calcd for C_24_H_26_N_2_O_2_S_2_, [M + H]^+^): 439.14, found: *M* = 439.04. Quantum yield in CHCl_3_ (*λ*_ex_ = 375 nm, *λ*_em_ = 437 nm) = 28%.

#### 3.2.4. 2,5-Bis(4-heptyloxyphenyl)thiazolo[5,4-*d*]thiazole ((HepOPh)_2_TTz)

Column chromatography using hexanes : CHCl_3_ (0.5 : 4.5) afforded 0.20 g (55%) of product. ^1^H-NMR (500 MHz, CDCl_3_, TMS, *δ*): 7.93 (d, *J* = 1.96 Hz, 2H), 6.98 (d, *J* = 2 Hz, 2H), 4.02 (t, *J* = 2 Hz, 2H), 1.81 (quint, *J* = 2.17 Hz, 2H), 1.47 (m, 2H), 1.30 (m, 6H), 0.90 (t, *J* = 3.21 Hz, 3H). UV-Vis *λ*_max_ (CHCl_3_, *ε* = M^−1^ cm^−1^): 375 nm (*ε* = 46 926). MALDI-TOF-MS (calcd for C_30_H_38_N_2_O_2_S_2_, [M + H]^+^): 523.24, found: *M* = 523.00. Quantum yield in CHCl_3_ (*λ*_ex_ = 373 nm, *λ*_em_ = 437 nm) = 25%.

### 3.3. Preparation of crystalline films

Two sets of symmetrically substituted D–A–D TTz polycrystalline blends were studied for color tuning and all-organic white light emission. The materials were chosen for a wide fluorescence tuning window and discernable PL emission signal. The mixtures were drop casted on pre-cleaned glass substrates the solvent was allowed to evaporate at room temperature.

#### Set 1: (MeOPh)_2_TTz and (PrOPh)_2_TTz

(MeOPh)_2_TTz was dispersed in ethanol and sonicated for 1 h to maximize the yellow emission originating from surface defects. 0.1 mg aliquots of the dispersion were dispensed in test tubes. (PrOPh)_2_TTz was dissolved in dichloromethane and added to the EtOH (MeOPh)_2_TTz suspension in varying weight proportions.

#### Set 2: (MeOPh)_2_TTz and (BuOPh)_2_TTz

(MeOPh)_2_TTz and (BuOPh)_2_TTz stock solutions were prepared in dichloromethane. To 0.1 mg of (MeOPh)_2_TTz, varying weight ratios of (BuOPh)_2_TTz were added and drop casted on glass substrates.

### 3.4. Computing CIE chromaticity coordinate

The emission of the TTz dyes was characterized by their location on a 2D color space known as chromaticity diagram. The chromaticity diagram is defined by the *x*, *y* coordinates defined as2
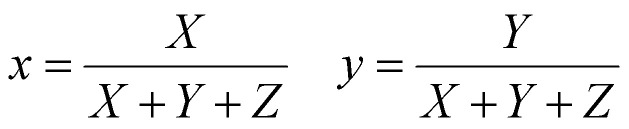
where the *X*, *Y*, and *Z* are computed by integrating the product of the emission spectrum of the dye and the appropriate color matching function over visible wavelengths:3
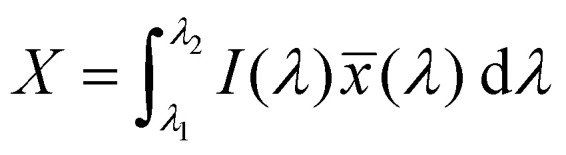
4
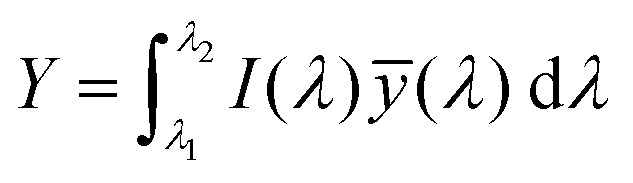
5
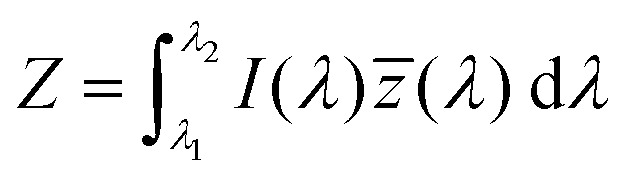
In eqn (3)–(5), *I*(*λ*) denotes the emission spectrum of the dye, *x̄*(*λ*), *ȳ*(*λ*), *z̄*(*λ*) are the CIE 1931 standard color matching functions, and the integration limits span visible wavelengths, *i.e. λ*_1_ = 380 nm and *λ*_2_ = 780 nm.^56^ We utilize the WPTherml software package^57^ to parse the experimental emission spectra and compute the CIE (*x*, *y*) coordinates through eqn (1) and (2), and the colour^58^ software package to render the CIE (*x*, *y*) coordinates as colors on the standard CIE chromaticity diagram and color gamut.

## 4. Conclusions

TTz-based materials have recently been used for a plethora of optical and optoelectronic applications. Studies utilizing TTz-based materials in the solid state mostly focus on polymeric materials. TTz-based materials have been seldom studied as a small molecule fluorescent dye in the solid state. Arguably this is because there is a significant gap of knowledge in correlating structure with photophysical properties in solid-state symmetrically substituted D–A–D TTz-based materials. In this study, we strategically functionalized TTz-based moiety with varying lengths of alkyl chain appendages. We have established that varying the alkyl chain length modulates the packing mode of these crystalline materials. The variation in packing modes is largely governed by a chorus of synergistic intermolecular non-covalent interactions. We have also established that modulation of crystal packing modes tunes the TTz photophysical properties such as their excitonic bandgap, photoluminescence emission, exciton lifetime, and lattice-phonon vibrational characteristics. The correlation between structure and photophysical properties of symmetrically substituted D–A–D TTz-based materials was then applied to fabricate crystalline blends. We utilized the multi-fluorochromic property of these materials to demonstrate that TTz-based crystals can be used for phosphor-converted color-tuning and white-light-emitting-diode applications. The cost effectiveness, solution processability and environment-friendly features of TTz-based materials present a compelling argument for their incorporation in solid-state photonic and fluorescence-based optical devices. This study will be crucial for the development of photonic materials and devices comprising of TTz-based small molecular fluorescent dyes.

## Abbreviations

TTz: Thiazolo[5,4-*d*]thiazole
(MeOPh)_2_TTz: 2,5-Bis(4-methoxyphenyl)thiazolo[5,4-*d*]thiazole
(PrOPh)_2_TTz: 2,5-Bis(4-propoxyphenyl)thiazolo[5,4-*d*]thiazole
(BuOPh)2TTz: 2,5-Bis(4-butoxyphenyl)thiazolo[5,4-d]thiazole
(HepOPh)_2_TTz: 2,5-Bis(4-heptyloxyphenyl)thiazolo[5,4-*d*]thiazole.

## Author contributions

Conceptualization: A. S., M. G. W.; formal analysis: A. S., T. A. S., L. T.; investigation: A. S., S. J., D. S. J., D. D., C. O. K., J. M. S., T. A. S.; methodology: A. S., J. M. S., T. A. S., J. J. F., M. G. W.; resources: D. S. J., T. A. S., J. J. F., M. G. W.; software: A. S., S. J., D. S. J., L. T., J. J. F., T. A. S.; supervision: M. G. W.; validation: M. G. W.; visualization: A. S., L. T., J. J. F., M. G. W.; writing – original draft: A. S.; writing – review and editing: A. S., M. G. W.

## Conflicts of interest

There are no conflicts to declare.

## Supplementary Material

MA-004-D3MA00686G-s001

MA-004-D3MA00686G-s002
