# Supplementary material for: Correlating structure and photophysical properties in thiazolo[5,4-d]thiazole crystal derivatives for use in solid-state photonic and fluorescence-based optical devices
Source: Mater Adv. 2023 Oct 20;4(23):6321–32. doi: 10.1039/d3ma00686g (PMC10680346; doi:10.1039/d3ma00686g)
Supplement: MA-004-D3MA00686G-s001 [file MA-004-D3MA00686G-s001.pdf]

Electronic Supplementary Material (ESI) for Materials Advances.
This journal is © The Royal Society of Chemistry 2023

# Correlating Structure and Photophysical Properties in Thiazolo[5,4-*d*]thiazole Crystal Derivatives for Use in Solid-State Photonic and Fluorescence-Based Optical Devices

*Abhishek Shibu, Sean Jones, P. Lane Tolley, David Diaz, Carly O. Kwiatkowski, Daniel S. Jones, Jessica M. Shivas, Jonathan J. Foley IV, Thomas A. Schmedake, and Michael G. Walter**

Department of Chemistry, University of North Carolina at Charlotte, Charlotte, NC, 28223,

*Michael.Walter@uncc.edu

**TABLE OF CONTENTS**


**Section 1: Spectra Characterization**

**Section 1.1: $^1$H-NMR Spectra……………………………………………………...3**

**Section 1.2: MALDI-TOF-MS……………………………………………………..7**

**Section 2: X-Ray Characterization**

**Section 2.1: Single Crystal Structure Data and Refinement…………………………8**

**Section 2.2: Correlation between Volume of Unit Cell and Alkyl Chain Length…....9**

**Section 2.3: Pictorial representation of interaction energies in TTz crystals……….10**

**Section 2.4: Intermolecular interaction energies in TTz crystals……………………11**

**Section 2.5: List of contacts from crystal packing data of $(C_nOPh)_2$TTz crystals….12**

**Section 2.6: Atom labelling sequence…………………………………………………13**

**Section 2.7: Distribution of intermolecular contacts to Hirshfeld surface area…….14**

**Section 2.8: Shape index of the Hirshfeld surfaces…………………………………...15**

**Section 2.9: Powder X-Ray Diffractograms of TTz Crystals………………………...16**

**Section 3: Photophysical Characterization**

**Section 3.1: DFT Simulated Raman Spectra.……………….……………...……17**

**Section 3.2: Lattice-Phonon Raman Spectra of TTz Crystals.….……………...……17**

**Section 3.3: Photostability of TTz Crystals…………………………………...……18**

**Section 4: References…………………………………………………………………...18**

**Section 1: Spectra Characterization**

**Section 1.1: *$^{1}$H-NMR Spectra***

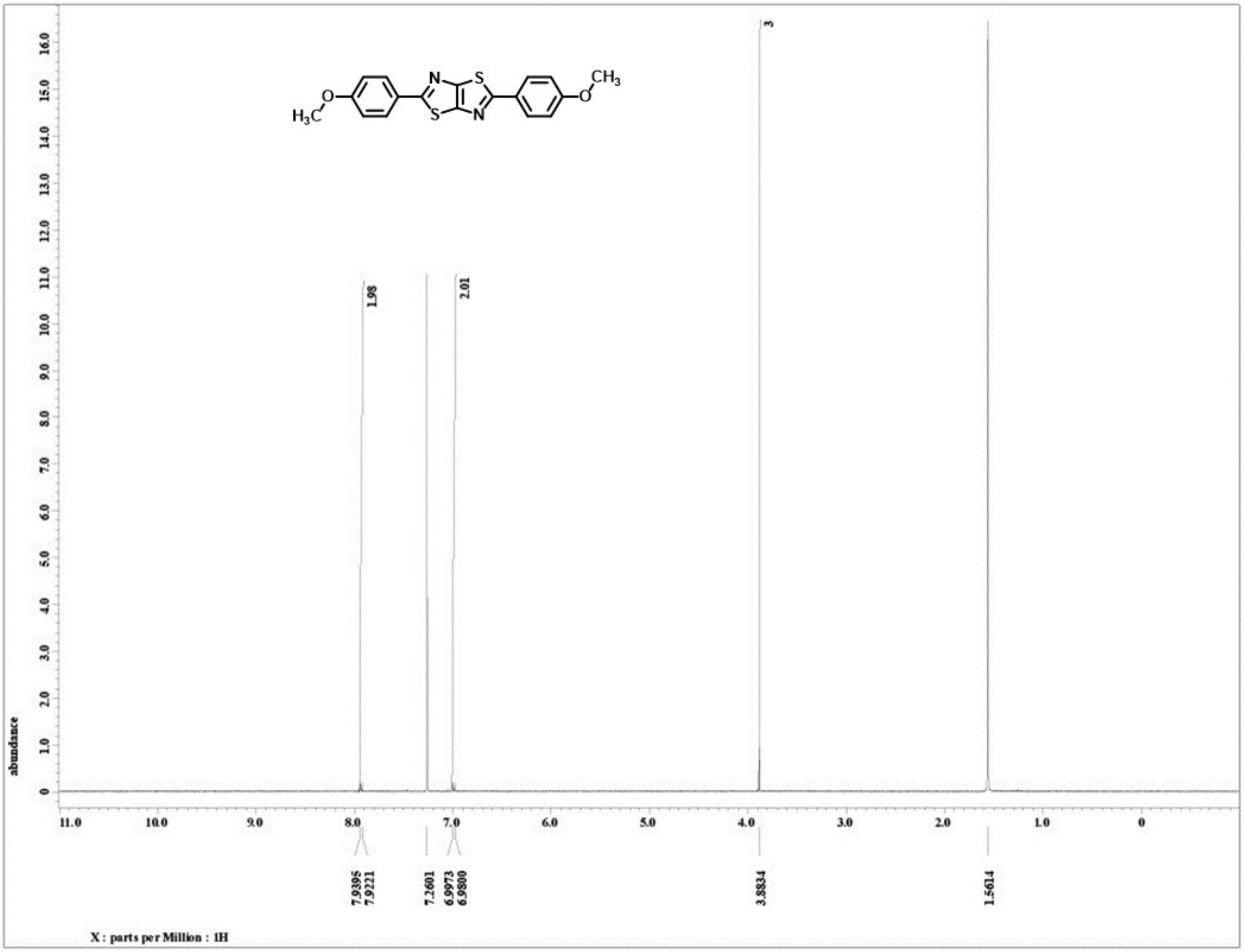

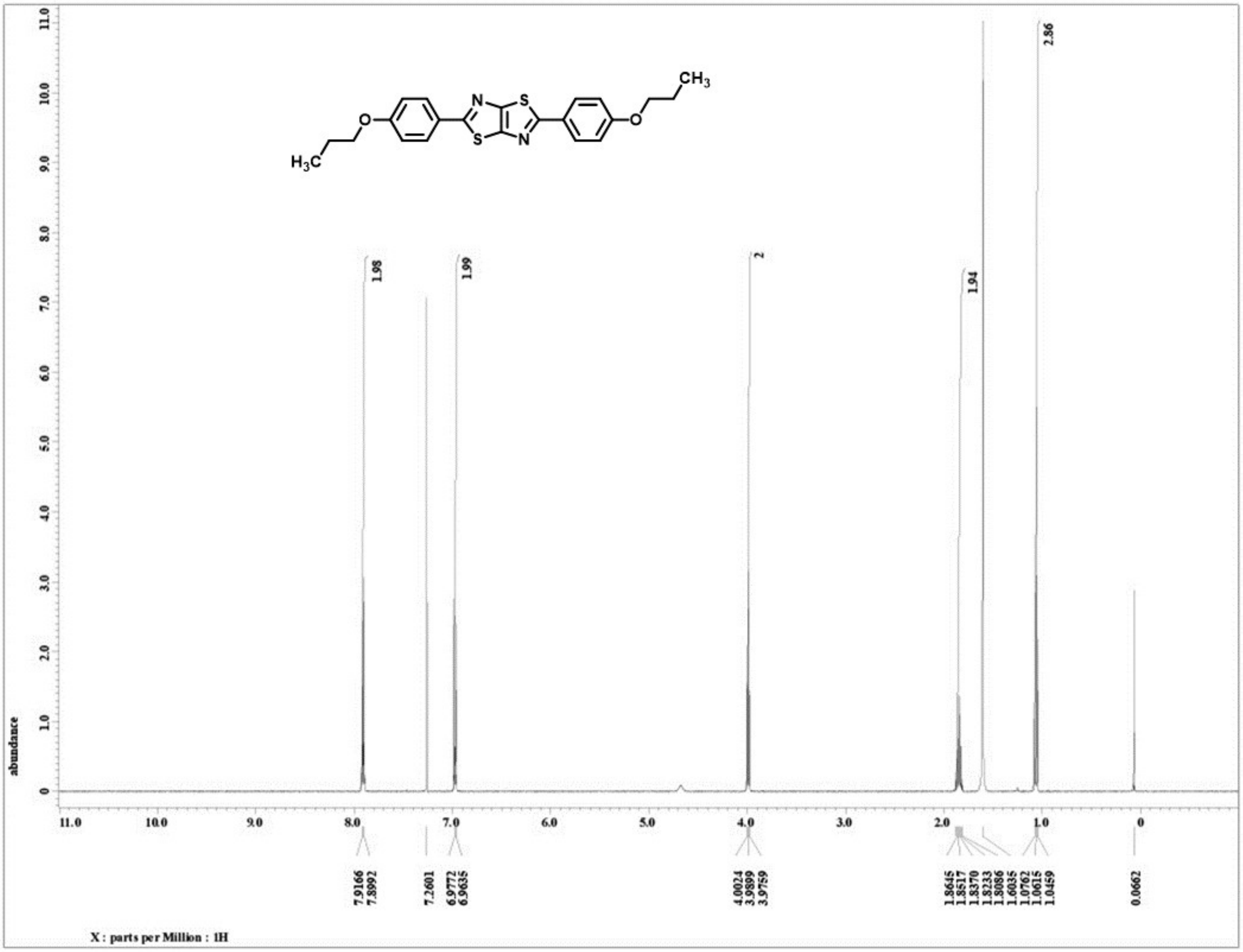
abundance
X : parts per Million : 1H
1.98
1.99
2
1.94
2.86
7.9166
7.8992
7.2601
6.9772
6.9635
4.0024
3.9899
3.9759
1.8645
1.8517
1.8370
1.8233
1.8086
1.6035
1.0762
1.0615
1.0459
0.0662

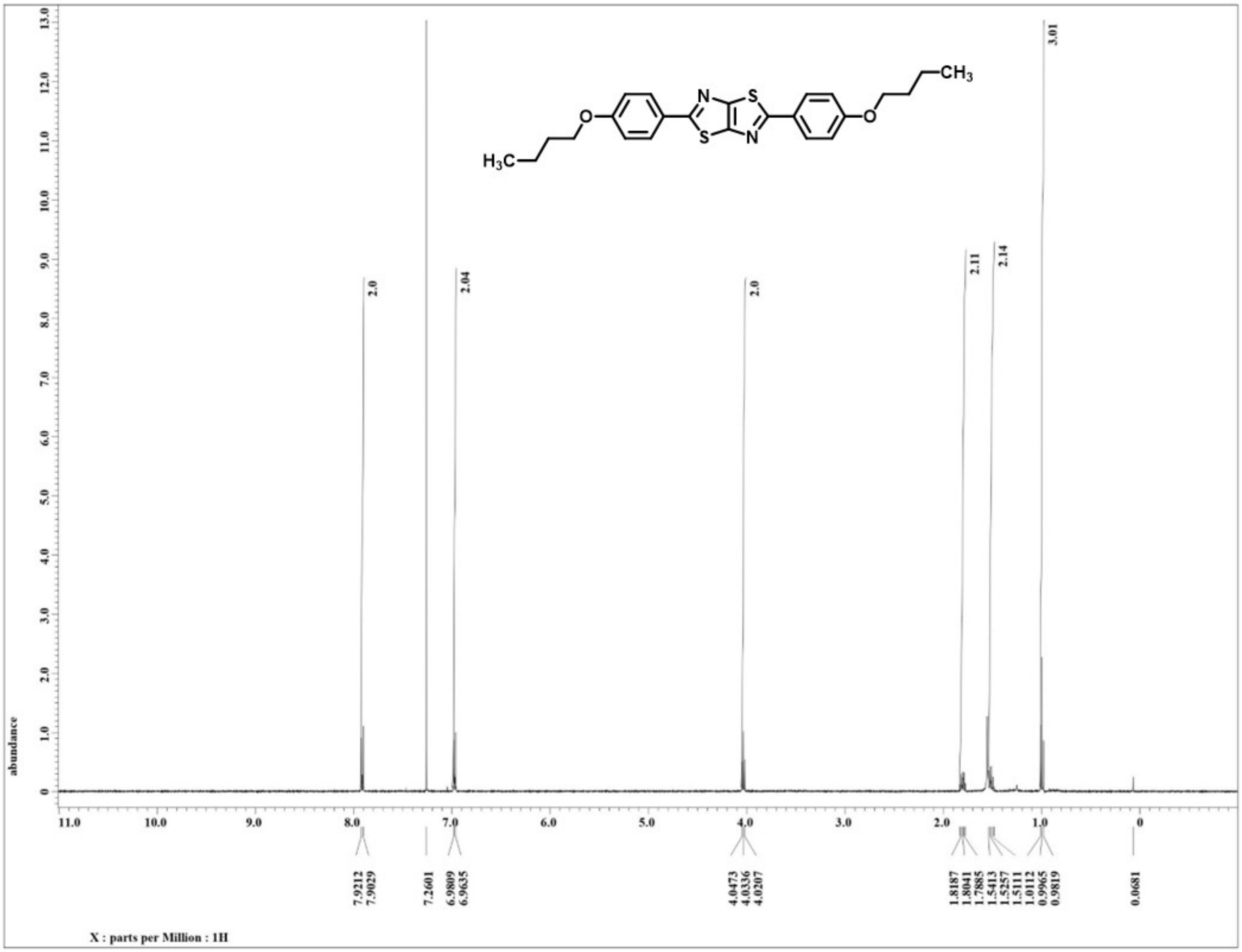

H3C
CH3
O
N
S
abundance
2.0
2.04
2.0
2.11
2.14
3.01
7.9212
7.9029
7.2601
6.9809
6.9635
4.0473
4.0336
4.0207
1.8187
1.8041
1.7885
1.5413
1.5257
1.5111
1.0112
0.9965
0.9819
0.0681
X : parts per Million : 1H

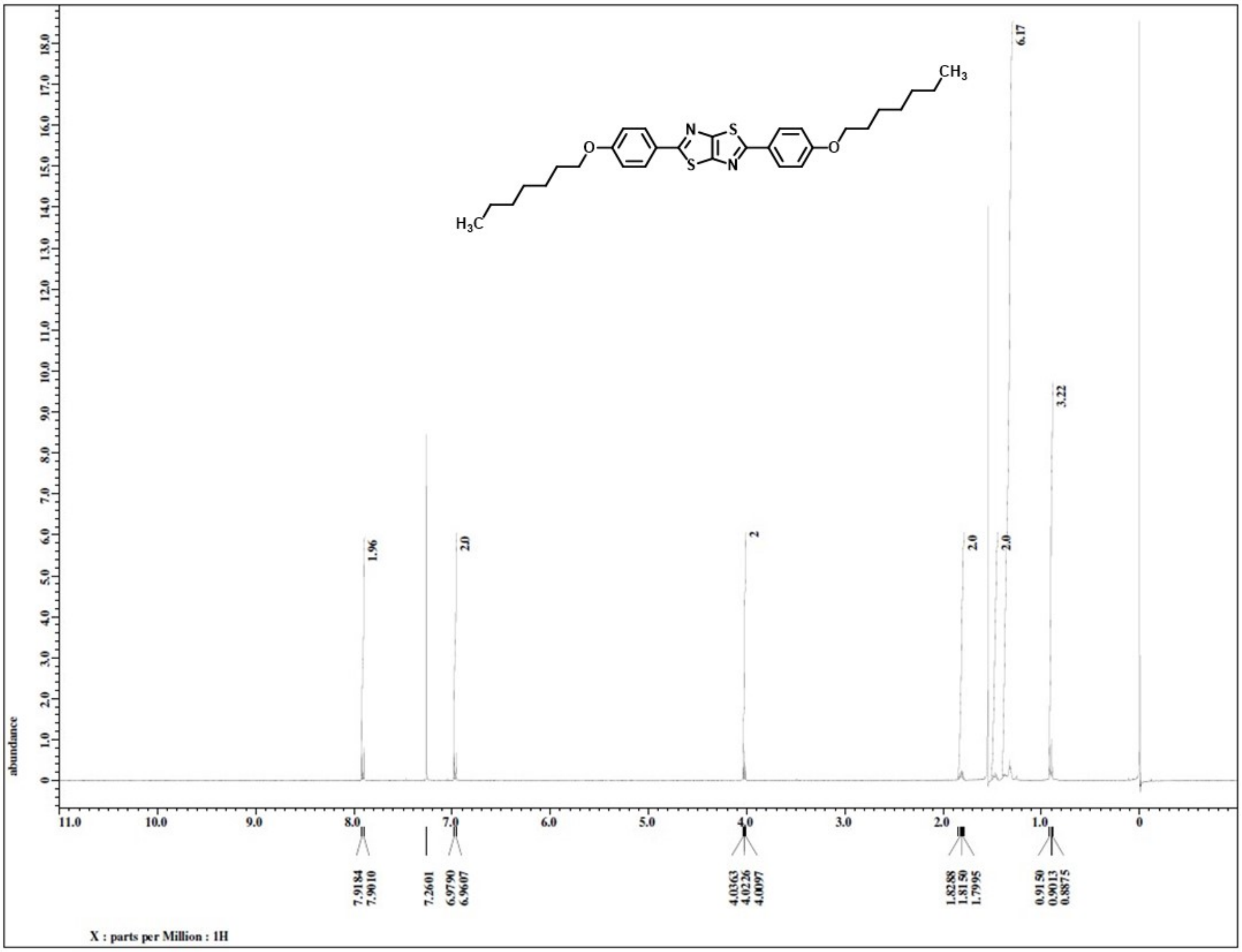

CH3
H3C
O
N
S
abundance
1.96
2.0
2
2.0
2.0
6.17
3.22
7.9184
7.9010
7.2601
6.9790
6.9607
4.0363
4.0226
4.0097
1.8288
1.8150
1.7995
0.9150
0.9013
0.8875
X : parts per Million : 1H

## Section 1.2: *MALDI-TOF-MS*

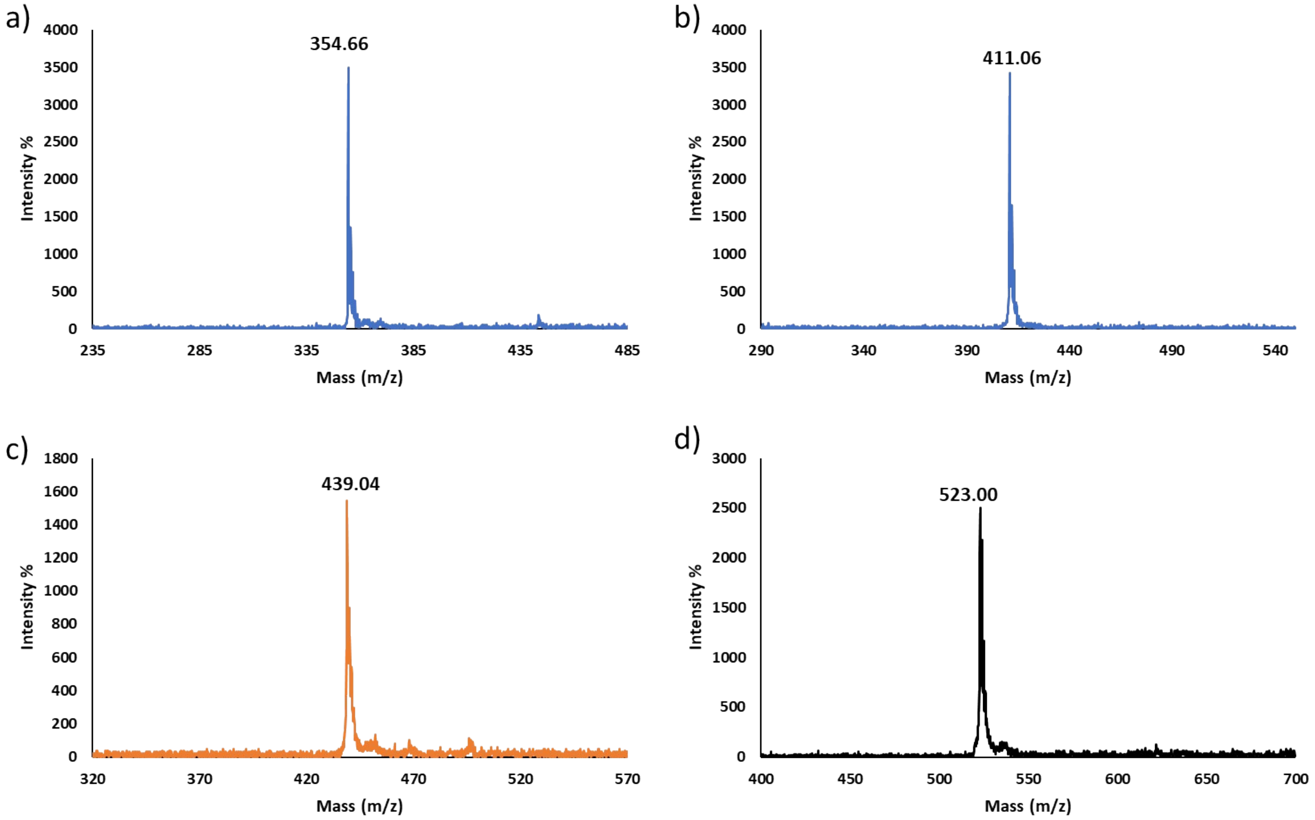


**Figure S1**: Maldi-TOF-MS Spectra of a) $(MeOPh)_2TTz$, b) $(PrOPh)_2TTz$, c) $(BuOPh)_2TTz$, and d) $(HepOPh)_2TTz$

## Section 2: X-Ray Characterization

## Section 2.1: *Single Crystal Structure Data and Refinement*

### Table S1: Summary of crystallographic data of dialkoxyphenyl TTzs

| Identification code | $(PrOPh)_2TTz$ | $(BuOPh)_2TTz$ | $(HepOPh)_2TTz$ |
|---|---|---|---|
| CSD Deposition Number | 2257069 | 2257068 | 2257067 |
| Empirical formula | $C_{22}H_{22}N_2O_2S_2$ | $C_{24}H_{26}N_2O_2S_2$ | $C_{30}H_{38}N_2O_2S_2$ |
| Formula weight | 410.564 | 438.618 | 522.781 |
| Temperature/K | 100.00(10) | 100.00(10) | 100.00(10) |
| Crystal system | monoclinic | triclinic | monoclinic |
| Space group | $P2_1/c$ | P-1 | $P2_1/c$ |
| a/Å | 16.2976(4) | 5.4441(5) | 31.0223(11) |
| b/Å | 7.46099(19) | 7.4759(7) | 7.1900(3) |
| c/Å | 7.9920(2) | 14.1023(13) | 6.0107(2) |
| α/° | 90 | 84.330(8) | 90 |
| β/° | 96.418(2) | 82.922(8) | 91.400(3) |
| γ/° | 90 | 70.044(8) | 90 |
| Volume/Å$^3$ | 965.71(4) | 534.39(9) | 1340.29(9) |
| Z | 2 | 1 | 2 |
| $\rho_{calc}$g/cm$^3$ | 1.412 | 1.363 | 1.295 |
| μ/mm$^{-1}$ | 2.669 | 2.446 | 2.032 |
| F(000) | 434.6 | 233.3 | 562.9 |
| Crystal size/mm$^3$ | 0.378 × 0.364 × 0.197 | 0.565 × 0.511 × 0.036 | 0.165 × 0.141 × 0.035 |
| Radiation | Cu Kα (λ = 1.54184) | Cu Kα (λ = 1.54184) | Cu Kα (λ = 1.54184) |
| 2Θ range for data collection/° | 10.92 to 133.34 | 12.62 to 133.6 | 8.56 to 133.84 |
| Index ranges | $-19 \le h \le 19, -7 \le k \le 8, -9 \le l \le 8$ | $-6 \le h \le 5, -8 \le k \le 6, -16 \le l \le 16$ | $-36 \le h \le 36, -8 \le k \le 7, -6 \le l \le 7$ |
| Reflections collected | 8742 | 4008 | 12196 |
| Independent reflections | 1698 [$R_{int}$ = 0.0335, $R_{sigma}$ = 0.0206] | 1873 [$R_{int}$ = 0.0374, $R_{sigma}$ = 0.0367] | 2371 [$R_{int}$ = 0.0772, $R_{sigma}$ = 0.0466] |
| Data/restraints/parameters | 1698/0/141 | 1873/0/137 | 2371/0/176 |
| Goodness-of-fit on F$^2$ | 1.075 | 1.075 | 1.103 |
| Final R indexes [I>=2σ (I)] | $R_1$ = 0.0300, $wR_2$ = 0.0733 | $R_1$ = 0.0366, $wR_2$ = 0.0945 | $R_1$ = 0.0744, $wR_2$ = 0.2012 |
| Final R indexes [all data] | $R_1$ = 0.0358, $wR_2$ = 0.0780 | $R_1$ = 0.0431, $wR_2$ = 0.1008 | $R_1$ = 0.0971, $wR_2$ = 0.2214 |
| Largest diff. peak/hole / e Å$^{-3}$ | 0.22/-0.28 | 0.40/-0.28 | 0.82/-0.43 |

**Section 2.2:** ***Correlation between Volume of Unit Cell and Alkyl Chain Length***

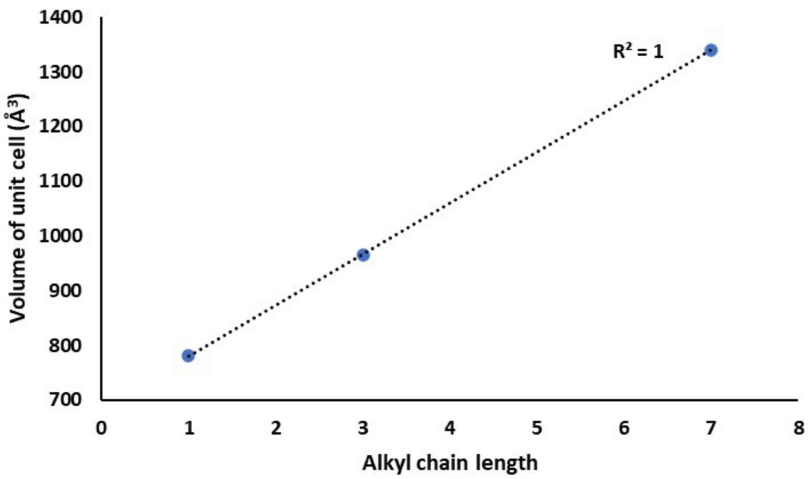


**Figure S2**: Crystals packed in monoclinic crystal system showed a perfect correlation between volume of unit cell and alkyl chain length.

## Section 2.3: Pictorial representation of interaction energies in TTz crystals

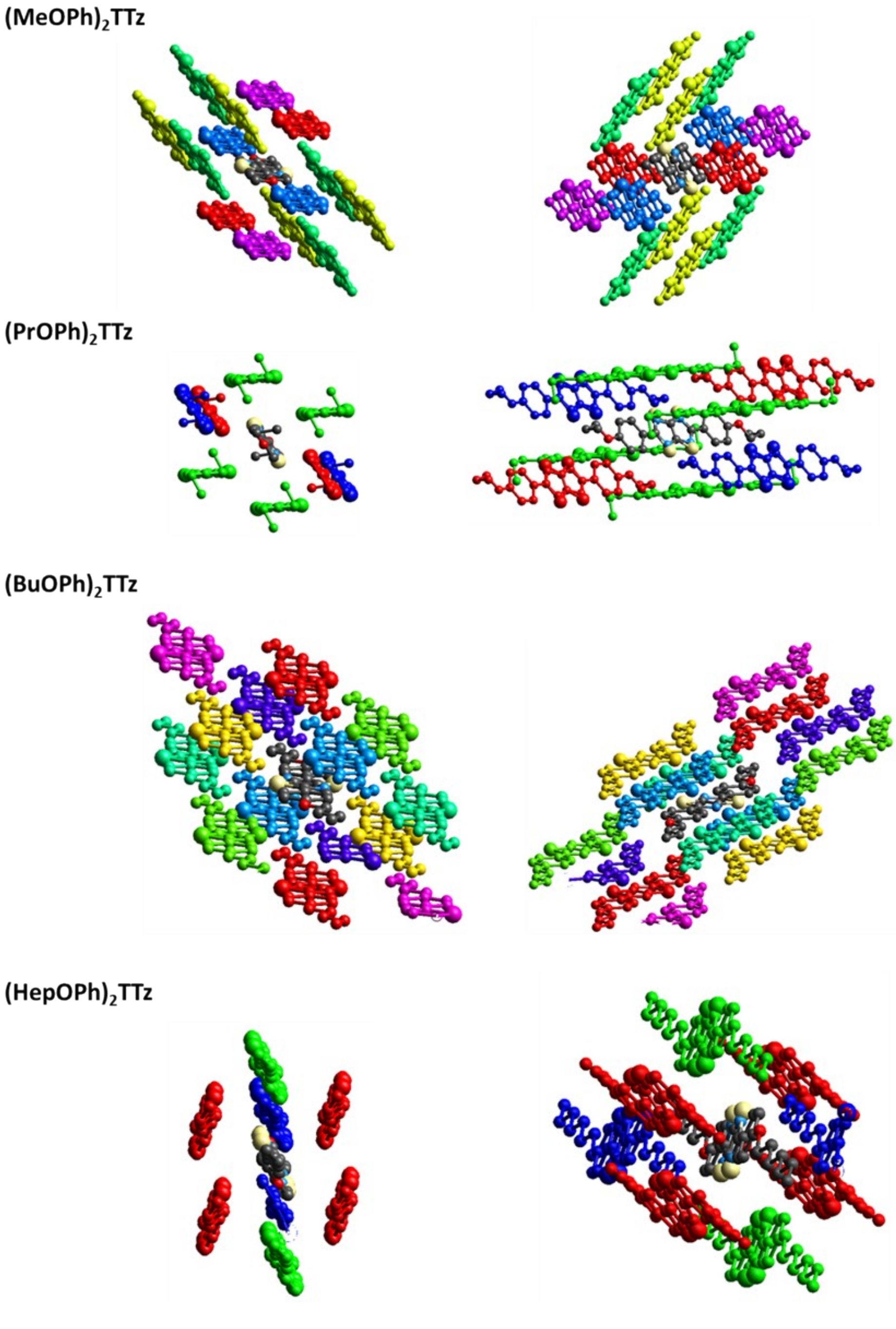


**Figure S3:** Color-coded pictorial representation of interaction energy in TTz crystals with respect to one central reference molecule.

## Section 2.4: Intermolecular interaction energies in TTz crystals

## Table S2: Details of intermolecular interaction energies in TTz crystals

| | **Pair label (*cf* Fig 3)** | **Orientation** | **Color** | **N** | **R (Å)** | **$E_{ele}$ (kJ/mol)** | **$E_{pol}$ (kJ/mol)** | **$E_{dis}$ (kJ/mol)** | **$E_{rep}$ (kJ/mol)** | **$E_{tot}$ (kJ/mol)** |
|---|---|---|---|---|---|---|---|---|---|---|
| **$(MeOPh)_2TTz$** | a) | Cofacial | Red | 2 | 3.98 | -6.98 | -2.44 | -80.74 | 36.34 | -53.70 |
| | b) | Orthogonal | Green | 4 | 10.67 | -11.73 | -1.48 | -25.69 | 14.28 | -24.60 |
| | c) | Parallel | Blue | 2 | 19.04 | -7.82 | -1.18 | -8.80 | 0.00 | -17.90 |
| | d) | Orthogonal | Yellow | 4 | 11.47 | -5.60 | -0.89 | -16.03 | 6.30 | -16.20 |
| | | Cofacial | Purple | 2 | 18.52 | 3.28 | -0.37 | -5.75 | 0.00 | -2.80 |
| **$(PrOPh)_2TTz$** | e) | Orthogonal | Green | 4 | 5.47 | -54.65 | -7.03 | -101.65 | 93.75 | -69.70 |
| | f) | Cofacial | Blue | 2 | 7.99 | -28.12 | -5.25 | -58.62 | 75.52 | -16.60 |
| | g) | Cofacial | Red | 2 | 18.94 | -0.11 | -0.07 | -8.97 | 0.00 | -9.20 |
| **$(BuOPh)_2TTz$** | h) | Cofacial | Red | 2 | 15.29 | -11.20 | -1.41 | -58.53 | 0.00 | -71.10 |
| | | Cofacial | Blue | 2 | 14.1 | 1.16 | -1.70 | -68.29 | 0.00 | -68.80 |
| | | Cofacial | Green | 2 | 15.96 | -29.81 | -1.26 | -27.35 | 0.00 | -58.50 |
| | | Cofacial | Teal | 2 | 14.48 | -10.36 | -2.00 | -33.01 | 0.00 | -45.40 |
| | | Cofacial | Yellow | 2 | 5.44 | -10.36 | -5.25 | -44.68 | 22.93 | -37.40 |
| | | Cofacial | Purple | 2 | 28.46 | 0.85 | 0.00 | -4.88 | 0.00 | -4.00 |
| | | Cofacial | Pink | 2 | 28.8 | 0.11 | 0.00 | -0.70 | 0.00 | -0.60 |
| **$(HepOPh)_2TTz$** | i) | Orthogonal | Red | 4 | 4.69 | -72.51 | 0.00 | -137.88 | 99.37 | -111.00 |
| | j) | Cofacial | Green | 2 | 6.01 | -79.06 | -7.62 | -78.39 | 110.44 | -54.70 |
| | | Cofacial | Blue | 2 | 33.54 | 0.21 | 0.00 | -7.06 | 0.00 | -6.80 |

R = distance between molecular centroids

$E_{tot}=E_{ele} + E_{pol} + E_{dis} + E_{rep}$

Where,

$E_{ele}$ = classical electrostatic energy of interaction between monomer charge distributions

$E_{rep}$ = exchange–repulsion energy

$E_{dis}$ = dispersion energy

$E_{pol}$ = Polarization energy

Since $k_BT$ = 2.5 kJ/mol and the error to which the sublimation enthalpy can be measured is approximately 5 kJ/mol for organic compounds, molecular pairs with $E_{tot} \sim$ 5 kJ/mol should be ignored.

## Section 2.5: List of contacts from crystal packing data of $(C_nOPh)_2TTz$ crystals

**Table S3: $(C_nOPh)_2TTz$ crystal packing data with emphasis on interchromophoric interactions. For atom labelling sequence *cf.* Figure S4.**

| | **Pair label (*cf* Fig 3)** | Orientation | Color | Interaction | Distance, (Å) | Angle, (degrees) | Frequency of Interaction | Number of molecules | Total |
|---|---|---|---|---|---|---|---|---|---|
| **$(MeOPh)_2TTz$** | a) | Cofacial | Red | $C_9$-H—$O_1$ | 2.935 | 149.48 | 2 | 2 | 4 |
| | | | | π-$\pi_{phenyl\ centroid}$ | 3.985 | | 2 | 2 | 4 |
| | b) | Orthogonal | Green | $C_5$-H—$S_1$ | 3.519 | 120.35 | 1 | 4 | 4 |
| | | | | $C_5$-H—$N_1$ | 2.964 | 178.48 | 1 | 4 | 4 |
| | | | | $C_9$—$S_1$ | 3.468 | 173.66 | 1 | 4 | 4 |
| | c) | | Yellow | $C_9$-H—$N_1$ | 2.912 | 161.62 | 1 | 4 | 4 |
| | | | | $C_7$-H—$S_1$ | 3.851 | 169.5 | 1 | 4 | 4 |
| | d) | Parallel | Blue | $C_9$-H—$O_1$ | 2.537 | 144.74 | 2 | 2 | 4 |
| **$(PrOPh)_2TTz$** | e) | Orthogonal | Green | $C_8$-H—$S_1$ | 3.839 | | 1 | 4 | 4 |
| | | | | $C_{10}$-H—$O_1$ | 3.249 | 127.93 | 1 | 4 | 4 |
| | | | | $C_{11}$-H—$O_1$ | 3.44 | 122.36 | 1 | 4 | 4 |
| | | | | $C_{10}$-H—$O_1$ | 3.922 | 135.47 | 1 | 4 | 4 |
| | | | | $C_2$—$S_1$ | 3.38 | | 1 | 4 | 4 |
| | | | | S—S | 3.488 | | 1 | 4 | 4 |
| | | | | $C_7$-H—$C_3$ | 2.88 | 136.06 | 1 | 4 | 4 |
| | | | | $S_1$—$C_3$ | 3.327 | | 1 | 4 | 4 |
| | | | | $C_4$—$S_1$ | 3.379 | | 1 | 4 | 4 |
| | f) | Cofacial | Blue | $C_7$-H—$N_1$ | 3.35 | 126.41 | 2 | 2 | 4 |
| | | | | $C_9$-H—$N_1$ | 2.986 | 72.88 | 2 | 2 | 4 |
| | | | | $C_8$—$S_1$ | 3.456 | | 2 | 2 | 4 |
| | g) | Cofacial | Red | $C_{11}$-H—$O_1$ | 3.799 | 142.93 | 2 | 2 | 4 |
| **$(BuOPh)_2TTz$** | h) | Cofacial | Red | $C_{12}$-H—$S_1$ | 3.266 | 123.54 | 2 | 14 | 28 |
| | | | | $C_9$-H—$O_1$ | 3.321 | 124.83 | 2 | 14 | 28 |
| | | | | $C_{12}$-H—$N_1$ | 3.255 | 170.66 | 2 | 14 | 28 |
| | | | | $C_{10}$-H—$\pi_{phenyl\ centroid}$ | 2.856 | 138.1 | 2 | 14 | 28 |
| | | | | $C_{11}$-H—$\pi_{phenyl\ centroid}$ | 3.591 | | 2 | 14 | 28 |
| | | | | $C_{12}$-H—$TTz_{centroid}$ | 3.601 | 142.8 | 2 | 14 | 28 |

| | | | | | | | | | |
|---|---|---|---|---|---|---|---|---|---|
| **(HepOPh)$_2$TTz** | i) | Orthogonal | Red | $C_{10}$-H—$O_1$ | 3.11 | 141.73 | 1 | 4 | 4 |
| | | | | $C_5$-H—$O_1$ | 3.893 | 147.75 | 1 | 4 | 4 |
| | | | | $C_{11}$-H—$O_1$ | 3.519 | 131.74 | 1 | 4 | 4 |
| | | | | $C_9$-H—$O_1$ | 3.016 | 144.94 | 1 | 4 | 4 |
| | | | | $C_4$-H—$S_1$ | 3.599 | 123.69 | 1 | 4 | 4 |
| | | | | $C_7$-H—$C_6$ | 2.826 | 124.37 | 1 | 4 | 4 |
| | | | | $S_1$—$C_2$ | 3.311 | | 1 | 4 | 4 |
| | | | | $S_1$—$C_1$ | 3.392 | | 1 | 4 | 4 |
| | | | | $C_4$-H—$C_3$ | 2.895 | 124.17 | 1 | 4 | 4 |
| | | | | $C_4$-H—$C_2$ | 2.892 | 144.2 | 1 | 4 | 4 |
| | j) | Cofacial | Green | $C_{11}$-H—O1 | 3.062 | 170.29 | 2 | 2 | 4 |
| | | | | $C_7$-H—H-$C_9$ | 2.345 | 157.81 | 2 | 2 | 4 |
| | | | | S—S | 3.118 | | 1 | 2 | 2 |

## Section 2.6: Atom labelling sequence

The atom labelling sequence of (HepOPh)$_2$TTz is shown in Figure S3. Truncated labelling sequence can be used for other TTz derivatives.

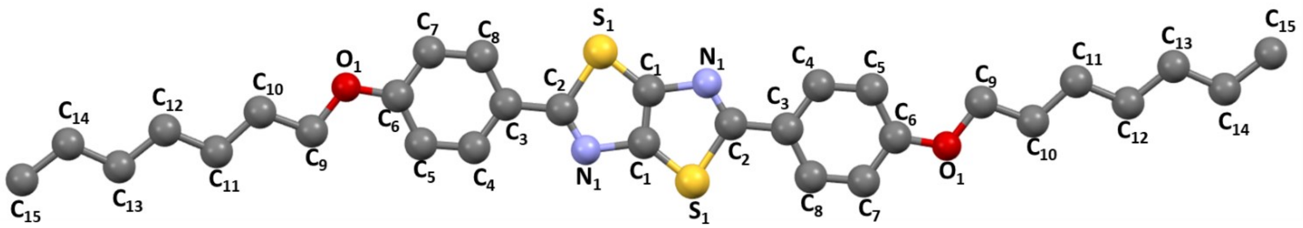


**Figure S4.** (HepOPh)$_2$TTz with labelled non-hydrogen atoms

**Section 2.7: Distribution of intermolecular contacts to Hirshfeld surface area**

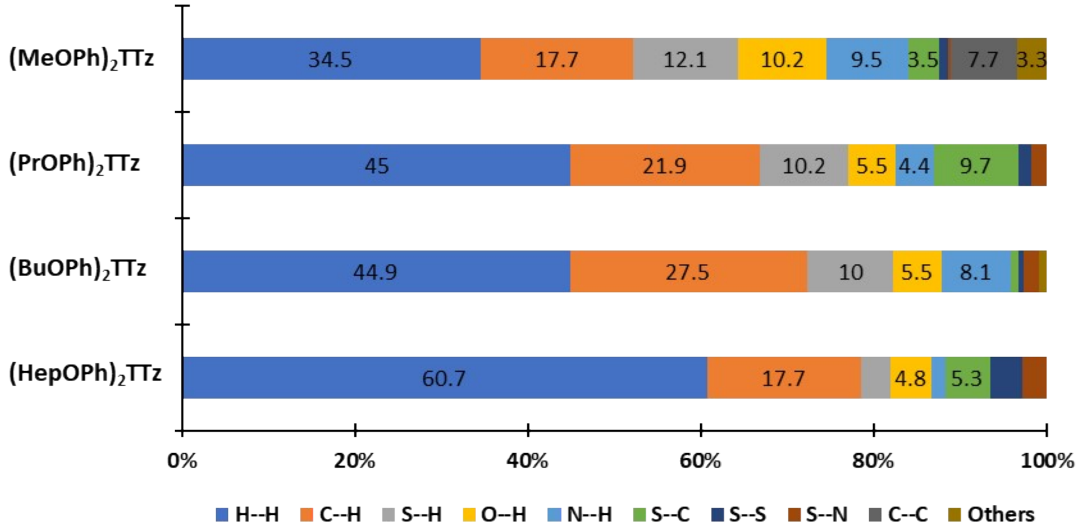


**Figure S5:** Relative contribution of the intermolecular contacts to the Hirshfeld surface area for (CnOPh)2TTz crystals.

## Section 2.8: Shape index of the Hirshfeld surfaces

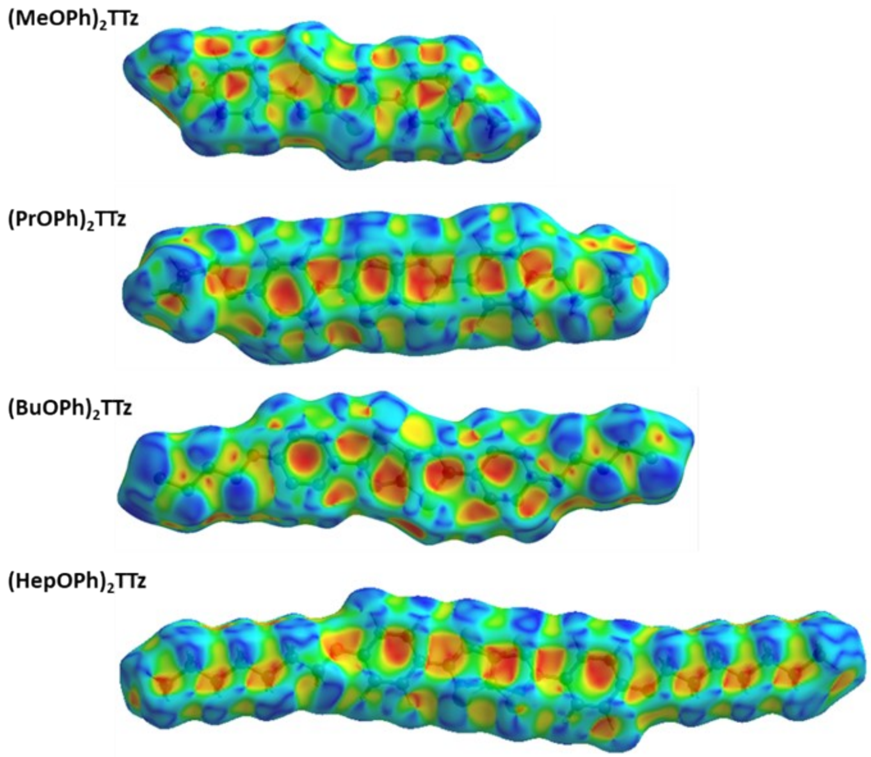


**Figure S6.** Shape index of the Hirshfeld surfaces of the $(C_nOPh)_2TTz$ crystals. π- π orbital overlap is represented by adjacent red and blue triangles on the Hirshfeld surface. The absence of adjacent red and blue triangles is indicative of lack of π- π orbital overlap.[1]

**Section 2.9:** ***Powder X-Ray Diffractograms of TTz Crystals***

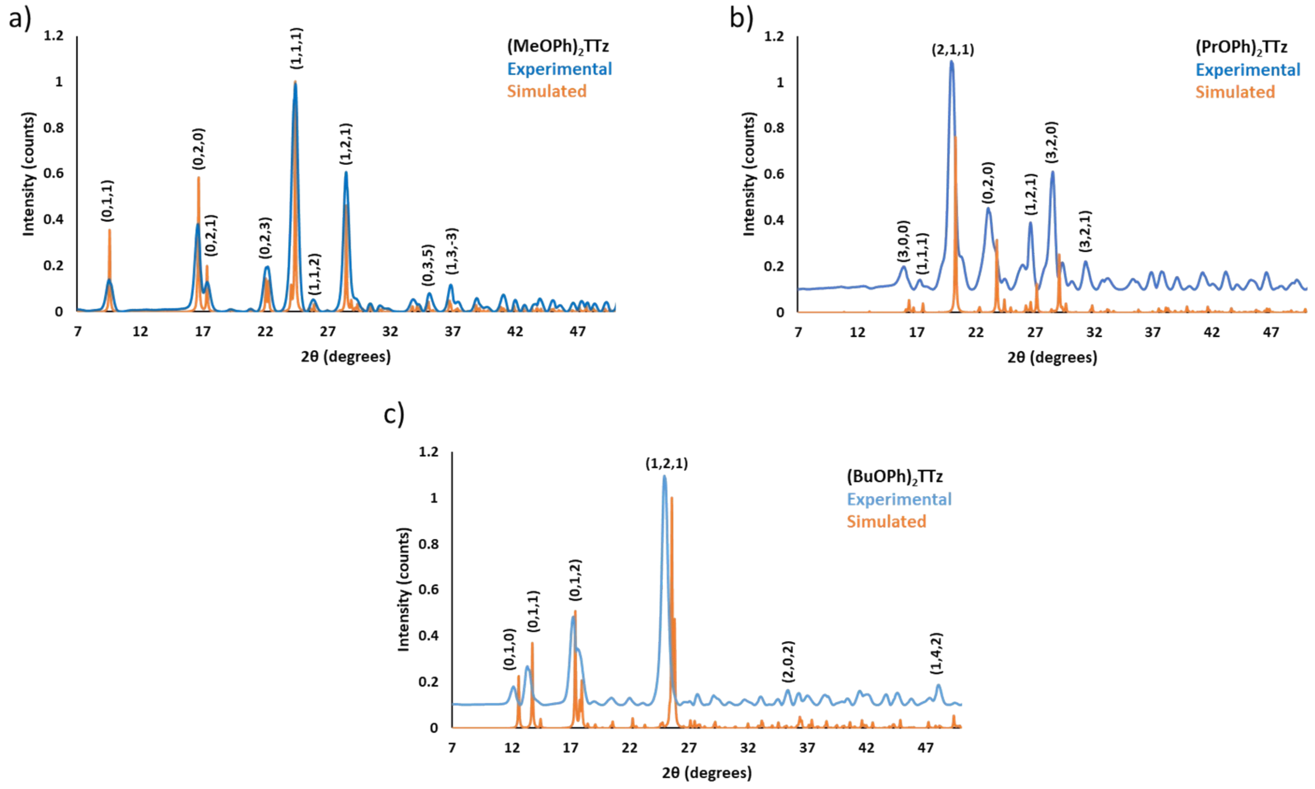


**Figure S7**: Comparison of experimental and simulated powder XRD patterns of a) $(MeOPh)_2TTz$, b) $(PrOPh)_2TTz$, c) $(BuOPh)_2TTz$.

**Section 3: Photophysical Characterization**

**Section 3.1: DFT Simulated Raman Spectra**

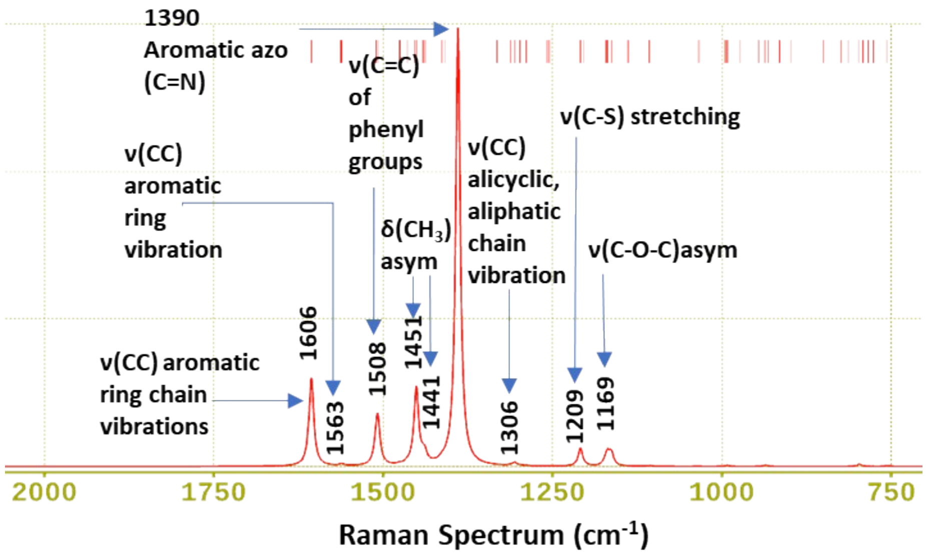


**Figure S8: DFT Simulated Raman Spectra with peak assignments.**

**Section 3.2: Lattice-Phonon Raman Spectra of TTz Crystals**

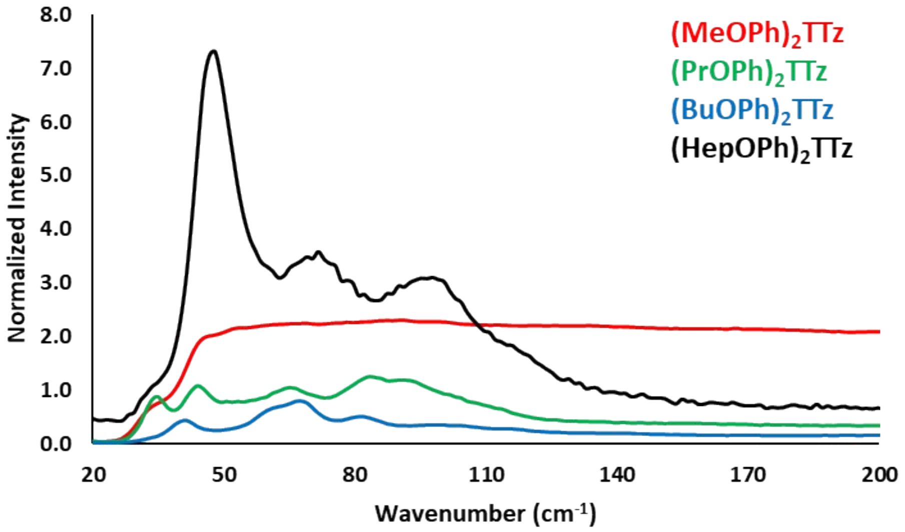


**Figure S9: Lattice-Phonon Raman Spectra of TTz Crystals.**

**Section 3.3:** ***Time Dependent Photostability Measurement of TTz Crystals***

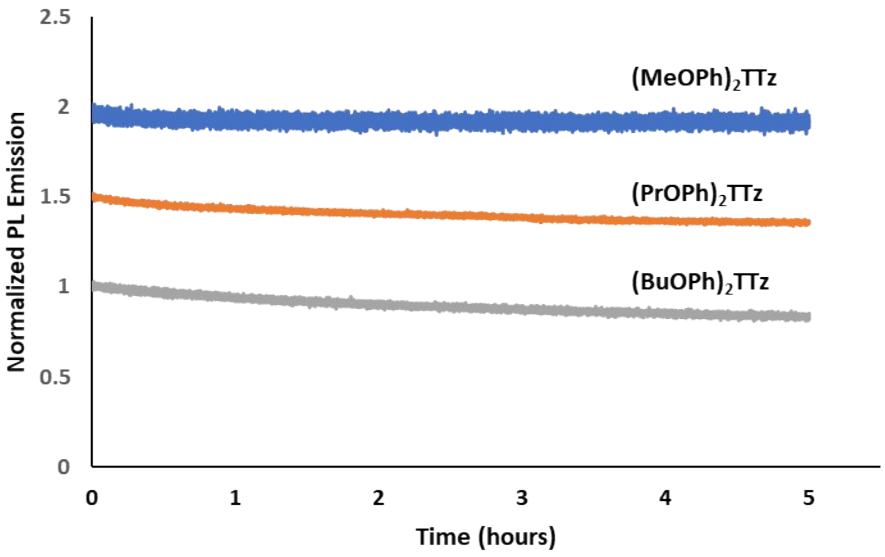


**Figure S10:** Time dependent photostability measurement of TTz Crystals under constant illumination by 400 nm in ambient conditions.

| **Material** | **Decrease in PL Intensity after 5 hours of illumination** |
|---|---|
| $(MeOPh)_2TTz$ | 8 % |
| $(PrOPh)_2TTz$ | 14 % |
| $(BuOPh)_2TTz$ | 15 % |

**Section 4: References**


(1) Shikhaliyev, N. Q.; Çelikesir, S. T.; Akkurt, M.; Bagirova, K. N.; Suleymanova, G. T.; Toze, F. A. Crystal structure and Hirshfeld surface analysis of (E)-1-(4-chlorophenyl)-2-[2, 2-dichloro-1-(4-fluorophenyl) ethenyl] diazene. *Acta Crystallographica Section E: Crystallographic Communications* **2019**, *75* (4), 465-469.
